# *Gl*Slt2 positively regulates *Gl*Myb-mediated cellulose utilization in *Ganoderma lucidum*

**DOI:** 10.1128/mbio.01812-25

**Published:** 2025-09-08

**Authors:** Zi Wang, Yefan Li, Hao Qiu, Zhouyu Li, Tianyu Ji, Ang Ren, Jing Zhu, Liang Shi, Mingwen Zhao, Rui Liu

**Affiliations:** 1 Key Laboratory of Agricultural Environmental Microbiology, Ministry of Agriculture and Rural Affairs, Department of Microbiology, College of Life Sciences, Nanjing Agricultural University 70578, Nanjing, Jiangsu, China; Cornell University, Ithaca, New York, USA

**Keywords:** *Gl*Slt2, *Gl*Myb, cellulose utilization, *Ganoderma lucidum*, cellulase

## Abstract

Fungal degradation of cellulose facilitates the sustainable harnessing of biosphere energy and carbon cycling. *Ganoderma lucidum* is one of the basidiomycetes with the largest number of hydrolytic enzymes in its genome. The mycelium of *G. lucidum* degrades cellulose through the production of substantial amounts of cellulase, enabling the absorption of carbon sources and nutrients essential for fruiting body development. The efficiency with which *G. lucidum* utilizes cellulose is a determinant of its growth rate. In this study, our findings revealed that the mitogen-activated protein kinase *Gl*Slt2 positively modulates cellulase activity and cellulose utilization. Furthermore, a yeast two-hybrid (Y2H) screening library found that *Gl*Slt2 interacts with *Gl*Myb, an R2R3-type MYB transcription factor. Further studies revealed that *Gl*Slt2 phosphorylates the S245 site of *Gl*Myb and that *Gl*Myb positively regulates cellulose utilization. *Gl*Myb directly binds to the [A/G] TTAC [G/C] [C/G] motif on the promoters of cellulase-related genes. The S245 site of *Gl*Myb promotes the binding of *Gl*Myb to the promoters of cellulase-related genes. Collectively, our findings highlight the mechanism by which *Gl*Slt2 positively regulates *Gl*Myb-mediated cellulose utilization. Enhancing cellulose utilization efficiency lays the foundation for the degradation of cellulose in agricultural and forestry waste and facilitates biomass conversion.

**IMPORTANCE:**

The proficient exploitation of cellulose is pivotal for fostering sustainable development, safeguarding the environment, and advancing economic prosperity and technological innovation. Paramount among these benefits is the reduction of reliance on fossil fuels. *Ganoderma lucidum*, a filamentous fungus, could effectively utilize cellulose from agricultural and forestry waste. Nevertheless, enhancing the efficiency of cellulose utilization from these by-products presents a formidable challenge that demands resolution. In our study, we discovered that GlSlt2 interacts with GlMyb and phosphorylates the S245 site of GlMyb. Further studies have revealed that GlSlt2 positively regulates GlMyb-mediated cellulose utilization. In summary, our findings unveil a sophisticated regulatory mechanism controlling cellulose utilization. These insights lay the foundation for biomass conversion and the biosphere carbon cycle.

## INTRODUCTION

Cellulose is the main component of plant cell walls and a globally abundant renewable carbohydrate (1, 2). Due to its renewable and biodegradable nature, cellulose is considered a potentially sustainable resource that can be used to produce a variety of products, including biofuels, bioplastics, paper, and other chemicals (3, 4). The conversion of cellulosic biomass requires powerful cellulases, which are primarily produced by filamentous fungi, such as *Trichoderma reesei, Penicillium oxalicum*, and *Aspergillus niger* (5–8). Furthermore, the degradation of cellulose by certain edible and medicinal macrofungi, including *Lentinula edodes*, *Agaricus bisporus*, and *Pleurotus ostreatus*, has garnered extensive research attention in recent years (9–11), suggesting that the degradation of cellulose by fungi is beneficial to the sustainable utilization of biosphere energy and the carbon cycle. Cellulase, an enzyme complex, comprises cellobiose hydrolases (CBHs), endoglucanases (EGs), and β-glucosidases (BGs), orchestrates the degradation of cellulose in a sequential manner: initially, CBHs break down crystalline cellulose to release cellobiose. Subsequently, EGs with high affinity for soluble cellulose derivatives further the degradation process. Ultimately, BGs catalyze the hydrolysis of cellobiose and oligosaccharides into glucose units, which are readily assimilated by fungi (12, 13). Numerous studies have demonstrated that various transcription factors, such as the carbon catabolite repressors CreA/Cre1 and ACE1, along with positive transcriptional activators XYR1/XlnR, CLR-1/ClrA, and CLR-2/ClrB, regulate cellulose degradation through transcription (12, 14–16). In addition, sugar transporters, including cellobiose, lactose, sorbose, and sophorose transport systems, participate in the degradation of cellulose by directly or indirectly influencing the transport of cellulase substrates (17, 18). However, screening for new cellulase regulators remains of great significance for perfecting the mechanism of cellulose degradation, promoting the application of biotechnology, and achieving the utilization of sustainable energy.

The mitogen-activated protein kinase (MAP kinase, MAPK) pathway, a pivotal signal transduction cascade in eukaryotes, comprises a trio of sequentially acting kinases: MAP kinase kinase kinase (MAPKKK), MAP kinase kinase (MAPKK), and MAP kinase (19, 20). This orchestrated phosphorylation relay efficiently propagates signals from initial sensory receptors through the kinase hierarchy to ultimately modulate the activity of downstream effectors, including transcription factors that govern a spectrum of physiological responses (21). Slt2, a member of the MAPK family, and the phosphorylation events it mediated plays a pivotal role in a myriad of biological processes. Slt2 facilitates arsenite efflux in *Saccharomyces cerevisiae* by phosphorylating the C-terminus of the aquaglyceroporin Fps1 (22). Slt2 phosphorylates Sir3, a component of the silent information regulator (SIR) complex, following treatment with rapamycin and chlorpromazine, preventing Sir3 from executing its silencing function in the subtelomeric regions and causing the derepression of certain cell wall stress-related genes (23). Slt2 phosphorylates the transcription factor Aft1, thereby negatively modulating its activity, which is crucial for iron homeostasis (24). Under cell wall stress conditions, activated Slt2 phosphorylates the transcription factor Rlm1, resulting in activation of Rlm1 transcriptional activity (25). The deletion of *Tmk2*, a Slt2 homolog, has been discovered to exert a substantial impact on cellulase activity in *T. reesei* (26). However, the underlying molecular mechanisms responsible for this effect remain to be fully deciphered.

The myeloblastosis (MYB) transcription factor family, distinguished by its conserved MYB DNA-binding domain, is prevalent among eukaryotic organisms. MYB transcription factors (TFs) have been implicated in a variety of biological processes, including secondary metabolism, cellular differentiation, and the growth and development of both plants and fungi (27). Furthermore, they have been identified as key players in the cellulose biosynthetic pathway. For example, the MYB72 TF was involved in the regulation of β-glucosidase BGLU42 in *Arabidopsis thaliana* (28). The rice MYB TF, *Os*MPS, plays a regulatory role in the expression of endoglucanase genes, specifically *OsGLU5* and *OsGLU14* (29). *Ff*Myb had emerged to participate in the degradation of cellulose by regulating the transcription level of cellulase-related genes in *Flammulina filiformis* (30). Despite these reports, it should be noted that research on the role of MYB transcription factors in cellulose utilization by filamentous fungi remains limited.

*Ganoderma lucidum*, a filamentous fungus, fulfills its growth and developmental requirements by secreting a suite of cellulases that enable the degradation of diverse agro-industrial cellulosic biomass materials (31, 32). Genomic sequencing of *G. lucidum* has revealed that it possesses an extensive array of wood-degrading enzymes, representing one of the largest such groups among the basidiomycetes (33). However, the mechanism of cellulose utilization in *G. lucidum* is still unclear. In our preceding investigations, we identified that signaling molecules, such as H_2_S and Ca^2+^, promote the cellulose utilization (34, 35). *Gl*Snf1, a component of the AMP-activated protein kinase (AMPK) family, regulates cellulose degradation by inhibiting *Gl*CreA during the utilization of cellulose (36). *Gl*Swi6, a transcription factor from the ASPES family, significantly increased the concentration of cytosolic Ca^2+^, thereby promoting the activities of cellulase (34). Considering the complexity of cellulose utilization, there remains a significant need to identify additional regulatory factors. Further exploration of additional proteins and molecular mechanisms within *G. lucidum* could aid in enhancing cellulose utilization, which is crucial for the production of biofuels and other bioenergy sources.

In this research, we identified that *Gl*Slt2, a key regulator, positively modulates cellulose utilization in *G. lucidum*. Furthermore, through a yeast two-hybrid (Y2H) screening library, we discovered *Gl*Myb, a transcription factor that interacts with *Gl*Slt2 and positively influences cellulase activity by binding to the promoter regions of cellulase-related genes (*CBH1*, *CBH3*, *EG1*, *EG3*, and *EG5*). Further research revealed that *Gl*Slt2 phosphorylates the S245 site of *Gl*Myb and positively regulates cellulose utilization. In summary, these findings have identified a novel regulatory factor for cellulase activity, offering new insights into the mechanisms of cellulose utilization.

## RESULTS

### *Gl*Slt2 functions as a positive regulator in cellulase activity and cellulose utilization in *G. lucidum*

**Fig 1:**
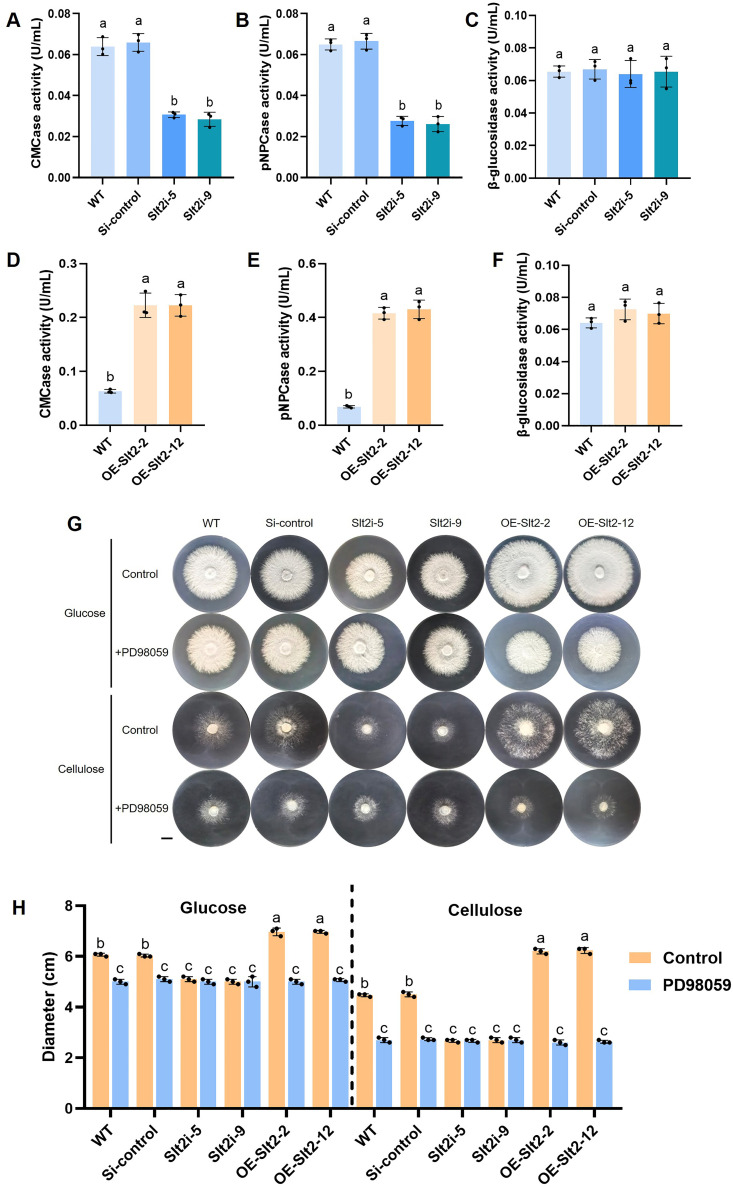
*Gl*Slt2 acts as a positive contributor to cellulase activity and cellulose utilization. The endocellulase (CMCase, **A**), exoglucanase (pNPCase, **B**), and β-glucosidase (**C**) activities were determined in the WT, Si-control, and *GlSlt2*-silenced strains. The endocellulase (**D**), exoglucanase (**E**), and β-glucosidase (**F**) activities in the WT and *GlSlt2*-overexpressing strains. (**G and H**) The growth phenotypes and hyphal diameter of the WT, Si-control, *GlSlt2*-silenced, and *GlSlt2*-overexpressing strains were assessed following 7 days of cultivation on glucose or sodium microcrystalline cellulose (CMC-Na) medium, either in the presence or absence of 20 µM PD98059 (scale bar = 1 cm). Control indicates that the culture medium does not contain PD98059. Data are presented as the mean ± SD (*n* = 3). Statistical significance is represented by different letters corresponding to *P* < 0.05 based on Tukey’s multiple range test.

To investigate the role of *Gl*Slt2 in cellulose utilization of *G. lucidum*, the previously constructed *GlSlt2*-silenced strains (Slt2i-5 and Slt2i-9), collected from the liquid culture medium with microcrystalline cellulose as the sole carbon source, were used to detect cellulase activity (37). We found that endocellulase activity (by approximately 55%) and exoglucanase activity (by approximately 58%) were significantly reduced in Slt2i-5 and Slt2i-9 strains compared to that in the WT and Si-control strains (Fig. 1A and B). However, there was no significant difference in β-glucosidase activity among these strains (Fig. 1C). To further confirm the biological function of *Gl*Slt2 in cellulose utilization, we generated *GlSlt2*-overexpressing strains using *Agrobacterium tumefaciens*-mediated transformation. And the OE-Slt2-2 and OE-Slt2-12 strains with increased transcript levels (by 4.1-fold and 4.5-fold, respectively) of *GlSlt2* relative to the control strain were used for further experiments (Fig. S1A and B). Then, the cellulase activity was examined in the WT and *GlSlt2*-overexpressing strains. Compared to the WT strain, the endocellulase and exoglucanase activities in the OE-Slt2-2 and OE-Slt2-12 strains were increased by 2.5-fold and 5-fold, respectively (Fig. 1D and E), while the β-glucosidase activity was similar among these strains (Fig. 1F). To demonstrate the potential influence of *Gl*Slt2 on cellulose utilization of *G. lucidum*, the WT, Si-control, *GlSlt2*-silenced, and *GlSlt2*-overexpressing strains were cultured for 7 days in medium containing either glucose or sodium carboxymethyl cellulose (CMC-Na) as the sole carbon source, and the growth phenotypes were observed. As shown in Fig. 1G and H, in the presence of glucose, the growth inhibition of the *GlSlt2*-silenced strains decreased by approximately 16% compared to the control strains. Conversely, the growth rate of the *GlSlt2*-overexpressing strains increased by approximately 14%. In the presence of cellulose, the *GlSlt2*-silenced strains exhibited a 42% reduction in hyphal diameter, while the *GlSlt2*-overexpressing strains showed a 27% increase. These results indicate that *Gl*Slt2 is involved in the growth of hyphae in the presence of glucose or cellulose, with a markedly stronger influence observed in cellulose-based growth medium.

PD98059 (HY-12028, MedChem Express), an inhibitor of Slt2 phosphorylation, functions by binding to the inactive form of the upstream MAPKK responsible for Slt2 activation (38). Previous studies have demonstrated that PD98059 at a concentration of 20 µM effectively suppresses the phosphorylation of *Gl*Slt2 in *G. lucidum* (39). Based on these findings, we treated these strains with 20 µM PD98059 and observed the growth of the hyphae. The results indicated that, regardless of whether in the presence of glucose or cellulose, the treatment with PD98059 inhibited the hyphal growth of both WT and Si-control strains, similar to the phenotype observed in the *GlSlt2*-silenced strains (Fig. 1G and H). However, the *GlSlt2*-silenced strains appeared unchanged in hyphal growth upon PD98059 treatment compared to their untreated strains (Fig. 1G and H). After the addition of PD98059 to the *GlSlt2*-overexpressing strains, the mycelial growth was inhibited, showing a phenotype similar to that observed in the treated WT strain. These results indicate that PD98059 may affect growth by inhibiting *Gl*Slt2.

**Fig 2:**
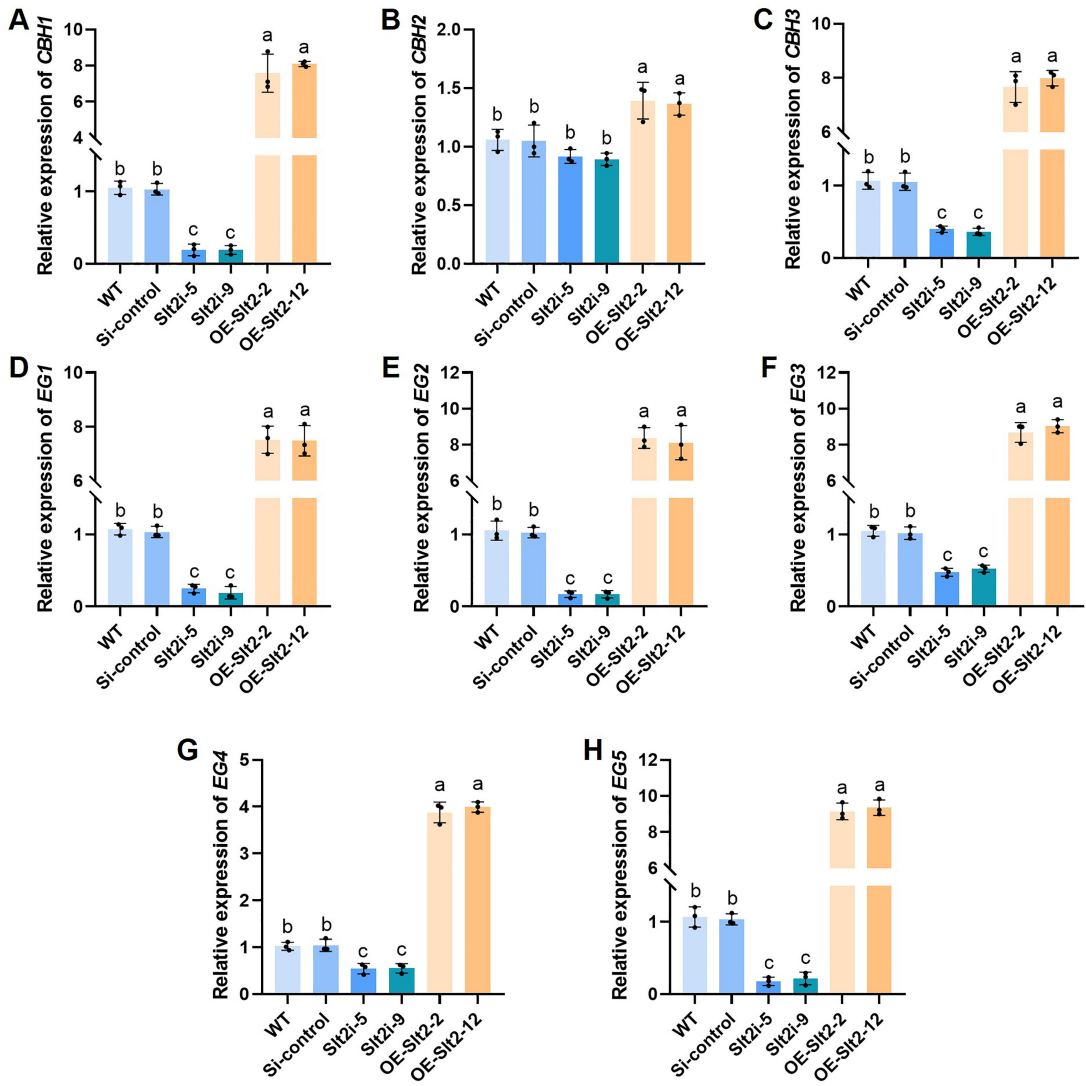
The relative expression levels of different cellulase-related genes. The relative expression levels of *CBH1* (**A**), *CBH2* (**B**), *CBH3* (**C**), *EG1* (**D**), *EG2* (**E**), *EG3* (**F**), *EG4* (**G**), and *EG5* (**H**) in the WT, Si-control, *GlSlt2*-silenced, and *GlSlt2*-overexpressing strains. Data are presented as the mean ± SD (*n* = 3). Statistical significance is represented by different letters corresponding to *P* < 0.05 based on Tukey’s multiple range test.

We previously revealed that there are three putative CBHs (CBH1, CBH2, and CBH3) and five EGs (EG1, EG2, EG3, EG4, and EG5) in *G. lucidum* (36). Next, we analyzed the *CBHs* and *EGs* gene expression in the WT, Si-control, *GlSlt2*-silenced strains, and *GlSlt2*-overexpressing strains collected from the liquid culture medium with microcrystalline cellulose as the sole carbon source. These cellulase-related genes were strongly upregulated in *GlSlt2*-overexpressing strains but significantly downregulated in *GlSlt2*-silenced strains compared with the control strains, while there was no significant difference in *CBH2* expression among these strains (Fig. 2A through H). These results demonstrate that *Gl*Slt2 contributes to the expression of cellulase-related genes.

To investigate whether a sufficiently high concentration of PD98059 could completely inhibit cellulase activity or the expression of cellulase-related genes, different concentrations of PD98059 were added to the liquid medium containing microcrystalline cellulose, followed by the collection of WT mycelia for cellulase activity and qRT-PCR analysis. It was found that the endocellulase and exoglucanase activities gradually decreased with the increase in PD98059 concentration, and when the concentration reached 20 µM, the activity no longer changed (Fig. S2A and B), while the β-glucosidase activity was similar among these concentrations (Fig. S2C). Similarly, qRT-PCR analysis revealed that, with the exception of *CBH2,* which showed no significant difference, the expression of other cellulase-related genes gradually decreased with the addition of PD98059 concentration, and remained unchanged when it reached 20 µM (Fig. S2D and E). These results indicate that high concentrations of PD98059 can significantly inhibit cellulase activity and expression levels, but cannot completely suppress them.

### *Gl*Slt2 interacts with *Gl*Myb *in vivo* and *in vitro*

**Fig 3:**
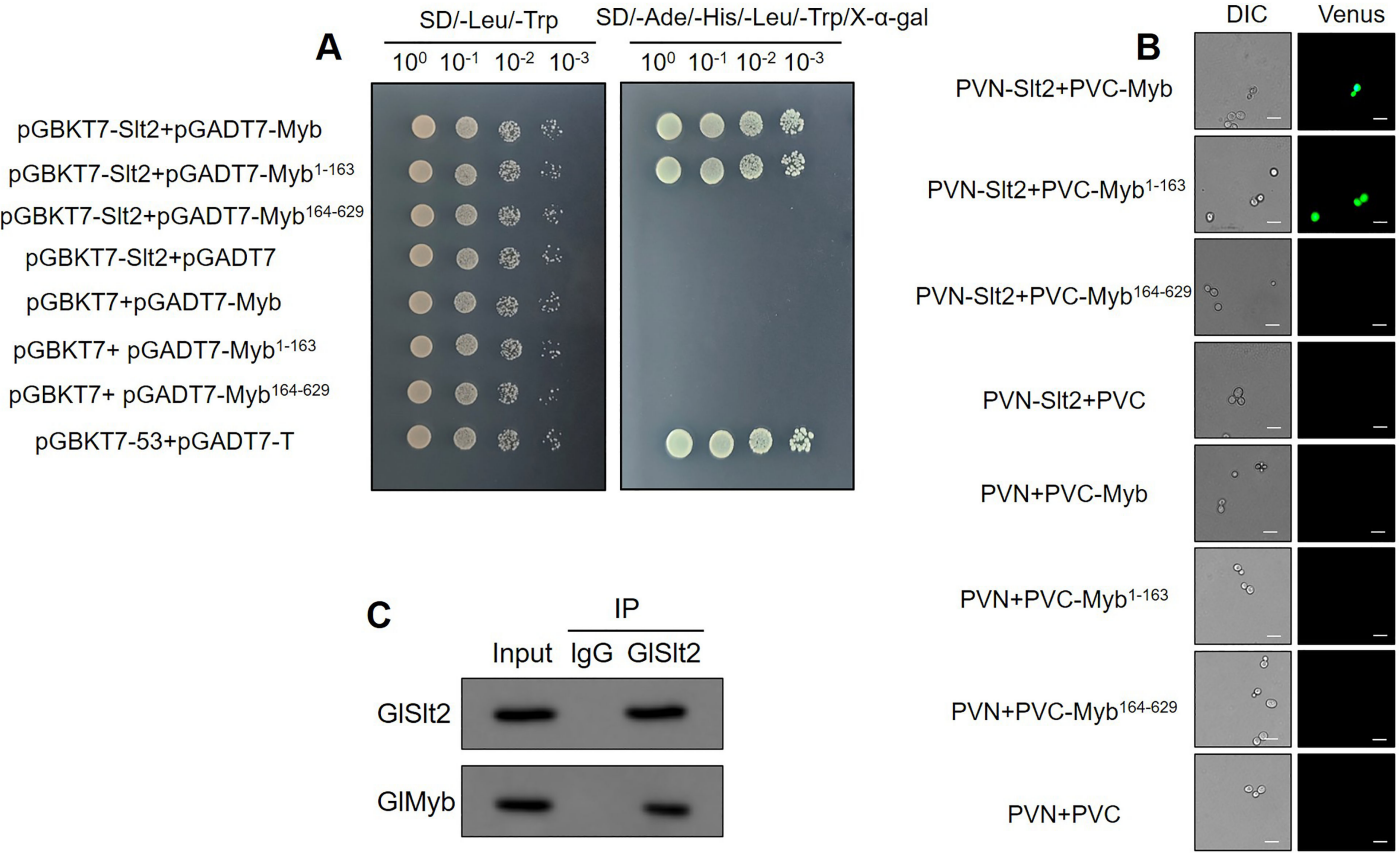
*Gl*Slt2 interacts with *Gl*Myb. (**A**) Y2H assay detection of the interaction between *Gl*Slt2 and *Gl*Myb. pGBKT7-Slt2, pGADT7-Myb, pGADT7-Myb^1–163^, and pGADT7-Myb^164–629^ were cotransformed into the Y2H strain. SD-Leu-Trp medium was used for testing successful mating, and SD-Ade-His-Leu-Trp/X-α-gal medium was used for testing interactions. The combination of pGBKT7-53 and pGADT7-T was used as the positive control. (**B**) BiFC verified the interaction between *Gl*Slt2 and *Gl*Myb. PVN-Slt2 containing the fragment of Venus-N, PVC-Myb, PVC-Myb^1–163^, and PVC-Myb^164–629^ containing the fragment of Venus-C were cotransformed into the SFY2620 yeast strain (scale bar = 100 µm). In addition, the recombinant plasmids PVN and PVC, PVN-Slt2, and PVC, as well as PVN and PVC-Myb, along with their truncated forms (PVC-Myb^1–163^ and PVC-Myb^164–629^), were cotransformed into yeast as negative controls. DIC, yeast cell morphology under the normal white field of view; Venus, a variant of GFP, yeast cell morphology under the green fluorescence. (**C**) Co-IP detection of the interaction between endogenous *Gl*Slt2 and *Gl*Myb. Immunoprecipitation of mycelial, collected from the liquid culture medium with microcrystalline cellulose as the sole carbon source, lysates with control rabbit IgG or anti-*Gl*Slt2 antibody. The IP products were detected by Western blotting with an anti-*Gl*Slt2 antibody to assess the accuracy of the experiment. In addition, the IP product was probed with an anti-*Gl*Myb antibody to specifically detect the interaction between *Gl*Slt2 and endogenous *Gl*Myb. Whole-cell extracts (Input) show the results of Western blot with anti-*Gl*Slt2 antibody and anti-*Gl*Myb antibody as controls.

To explore the molecular mechanism of *Gl*Slt2 regulating cellulose utilization, we conducted a Y2H assay and identified 13 potentially interacting proteins (Table S1). Among the putative interactors, *Gl*30464 (*Gl*Myb), an R2R3-type Myb transcription factor, came to our attention because *Ff*Myb had emerged to participate in the degradation of cellulose by regulating the transcription level of cellulase-related genes in *F. filiformis* (30). To determine whether *Gl*Slt2 interacts with *Gl*Myb, we carried out a Y2H assay and found that *Gl*Slt2 indeed interacted with *Gl*Myb in yeast (Y2H) cells, and *Gl*Slt2 strongly interacted with the fragment of *Gl*Myb containing a Myb-like DNA-binding domain (Myb^1–163^; Fig. 3A). Moreover, the bimolecular fluorescent complementary (BiFC) assay was performed. The results suggested that yeast strain SFY2620 cotransformed with PVN-Slt2 and full-length PVC-Myb or PVC-Myb^1–163^ fusion vectors exhibited strong fluorescence signals, whereas the yeast strain co-expressing PVN-Slt2 and PVC-Myb^164–629^ did not produce any detectable signals (Fig. 3B). These findings suggest that *Gl*Slt2 interacts with *Gl*Myb *in vitro*, with the binding site located at *Gl*Myb^1–163^.

To further confirm the interaction between endogenous *Gl*Slt2 and *Gl*Myb, *G. lucidum* mycelium was collected from the liquid culture medium with microcrystalline cellulose as the sole carbon source, followed by an immunoprecipitation assay. After immunoprecipitation with anti-*Gl*Slt2 or IgG beads, the IP product was treated with anti-*Gl*Slt2 antibody and subjected to Western blot analysis to assess the accuracy of the experiment. Meanwhile, the IP product was also treated with anti-*Gl*Myb antibody to examine the interaction with endogenous *Gl*Slt2. The results reveal that endogenous *Gl*Slt2 forms a physical complex with endogenous *Gl*Myb in *G. lucidum* (Fig. 3C). Collectively, these data demonstrate that *Gl*Slt2 physically interacts with *Gl*Myb *in vivo* and *in vitro*.

### *Gl*Slt2 phosphorylates *Gl*Myb *in vivo* and *in vitro*

**Fig 4:**
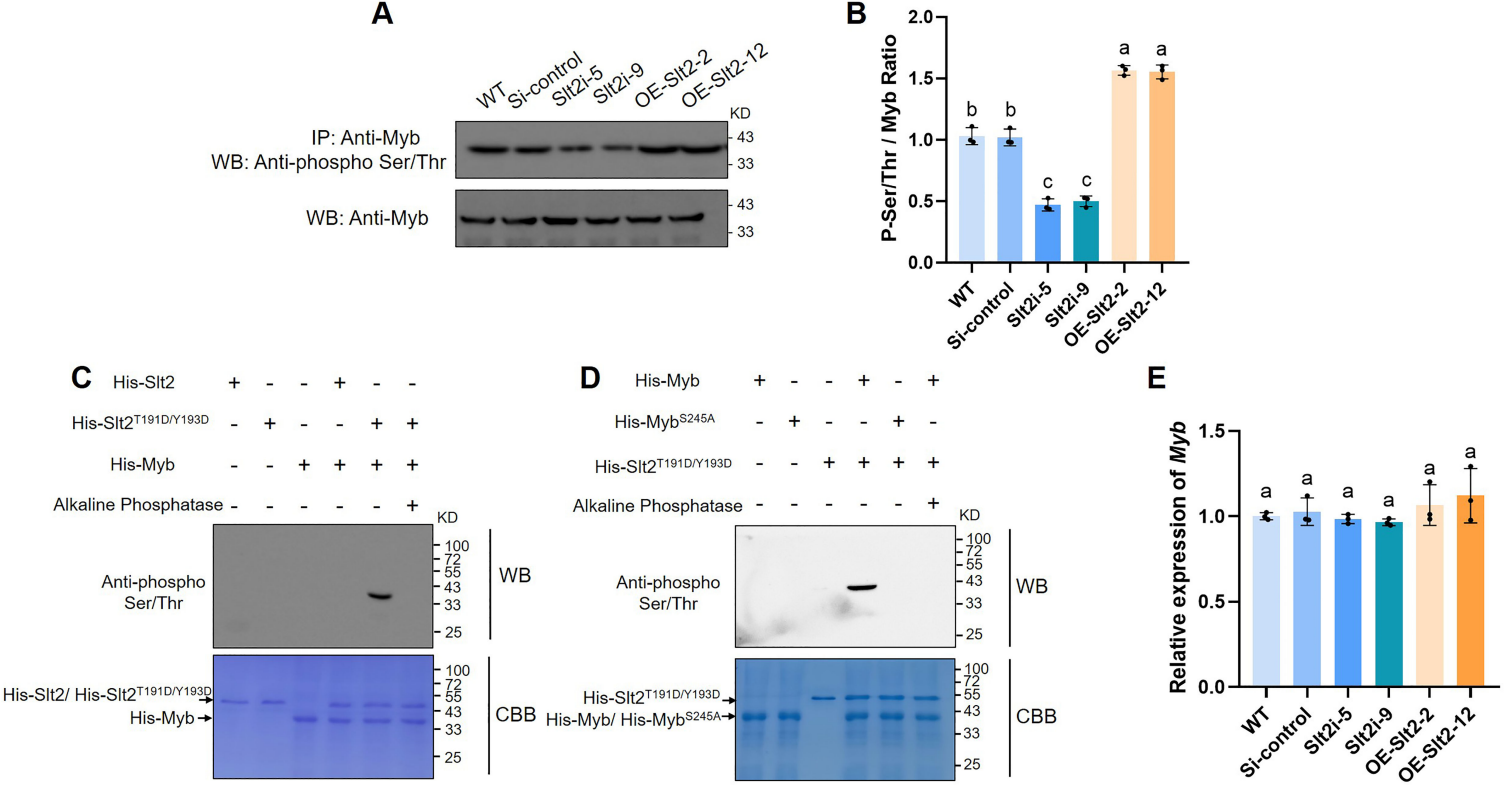
*Gl*Slt2 phosphorylates *Gl*Myb *in vivo* and *in vitro*. (**A**) Detection of the phosphorylation level of *Gl*Myb in the WT, Si-control, *GlSlt2*-silenced, and *GlSlt2*-overexpressing strains collected from the liquid culture medium with microcrystalline cellulose as the sole carbon source by immunoblotting using an anti-phospho Ser/Thr antibody. (**B**) P-Ser/Thr/Myb ratio in panel (**A**). (**C**) *Gl*Slt2 phosphorylates *Gl*Myb *in vitro*. The purified His-Slt2 or His-Slt2^T191D/Y193D^ fusion protein was incubated with His-Myb in phosphorylation buffer for 30 min at 30°C. As for calf intestinal alkaline phosphatase (CIAP) treatment, protein extracts were incubated with CIAP (37°C, 30 min) before SDS-PAGE separation and immunoblot analysis. (**D**) *Gl*Slt2 phosphorylates the S245 site of *Gl*Myb *in vitro*. The purified His-Myb or His-Myb^S245A^ fusion protein was incubated with His-Slt2^T191D/Y193D^ in phosphorylation buffer for 30 min at 30°C. As for CIAP treatment, protein extracts were incubated with CIAP (37°C, 30 min) before SDS–PAGE separation and immunoblot analysis. A gel stained with Coomassie Brilliant Blue was used as a loading control. + and – denote the presence and absence of proteins, respectively, in each sample. (**E**) Transcriptional levels of *GlMyb* in the WT, Si-control, *GlSlt2*-silenced, and *GlSlt2*-overexpressing strains were collected from a liquid medium with microcrystalline cellulose as the sole carbon source. Data are presented as the mean ± SD (*n* = 3). Statistical significance is represented by different letters corresponding to *P* < 0.05 based on Tukey’s multiple range test.

As a protein kinase, its primary function is to phosphorylate interacting proteins. The phosphorylation status of *Gl*Myb protein was assayed by immunoblotting in the control, *GlSlt2*-silenced strains, and *GlSlt2*-overexpressing strains collected from the liquid culture medium with microcrystalline cellulose or glucose as the sole carbon source (Fig. 4A; Fig. S3). The results indicate that in the presence of cellulose, the phosphorylation of *Gl*Myb immunoprecipitated with *GlSlt2*-silenced strains (52%) was significantly lower than those in the WT and Si-control (Fig. 4B). On the contrary, the phosphorylation levels of *Gl*Myb immunoprecipitated with a *GlSlt2*-overexpressing strains increased by 50%, confirming that *Gl*Slt2 phosphorylates *Gl*Myb *in vivo*. In the presence of glucose, the phosphorylation level of *Gl*Myb did not show significant differences among these strains (Fig. S3A and B). In previous studies, it was reported that *Gl*Slt2 is involved in responding to cell wall stress in *G. lucidum* (37). To detect whether *Gl*Slt2 phosphorylates *Gl*Myb under cell wall stress, the control, *GlSlt2*-silenced strains, and *GlSlt2*-overexpressing strains collected from glucose-based medium containing CFW or CR (Fig. S3C and E). It was found that *Gl*Slt2 did not affect the phosphorylation of *Gl*Myb in the presence of CFW or CR (Fig. S3D and F). These results suggest that *Gl*Slt2 may not be involved in responding to cell wall stress through the phosphorylation of *Gl*Myb.

In addition, the recombinant proteins pColdI-*Gl*Slt2 and pColdI-*Gl*Myb were expressed and purified, followed by *in vitro* phosphorylation assays (Fig. 4C). Surprisingly, the results showed that after co-incubation with *Gl*Slt2 and *Gl*Myb, no signal was detected by immunoblotting using an anti-phosphoSer/Thr antibody (Fig. 4C, *lane 4*). Previous studies have demonstrated the importance of threonine (T) and tyrosine (Y) in the TGY motif for Slt2 activity (40, 41). Therefore, the T191 and Y193 sites of *Gl*Slt2 were mutated, replacing them with aspartic acid (D, Slt2^T191D/Y193D^) to mimic the active form, and we observed evidence that *Gl*Slt2^T191D/Y193D^ could phosphorylate *Gl*Myb (Fig. 4C, *lane 5*), which was strongly inhibited by the calf intestinal alkaline phosphatase (CIAP, Fig. 4C, *lane 6*). These immunoblotting results showed that *Gl*Slt2^T191D/Y193D^ phosphorylates *Gl*Myb *in vitro*. To further explore the phosphorylation sites of *Gl*Myb by *Gl*Slt2, the Scansite software (https://scansite4.mit.edu) was utilized. Computational analysis identified a single putative phosphorylation site, S245, on the *Gl*Myb protein sequence (Fig. S4). To verify this putative phosphorylation site, the S245 site of *Gl*Myb was mutated, replacing it with alanine (A, Myb^S245A^) to mimic the nonphosphorylation. Subsequently, the recombinant protein pColdI-*Gl*Myb^S245A^ was induced and purified, and *in vitro* phosphorylation assay was performed again (Fig. 4D). It was found that, compared to the control (Fig. 4D, *lane 4*), the addition of *Gl*Myb^S245A^ did not detect any signal (Fig. 4D, *lane 5*). This result indicates that the S245 site of *Gl*Myb is indeed the phosphorylation site of *Gl*Slt2.

In addition, the expression of *GlMyb* was analyzed in the control, *GlSlt2*-silenced strains, and *GlSlt2*-overexpressing strains collected from the liquid culture medium with microcrystalline cellulose as the sole carbon source. Notably, there was no significant difference in *GlMyb* transcript levels among these strains (Fig. 4E). The above findings suggest that in the presence of cellulose, *Gl*Slt2 phosphorylates *Gl*Myb, implying a role in protein modification rather than in transcriptional regulation.

### *Gl*Myb positively regulates cellulase activity and cellulose utilization

**Fig 5:**
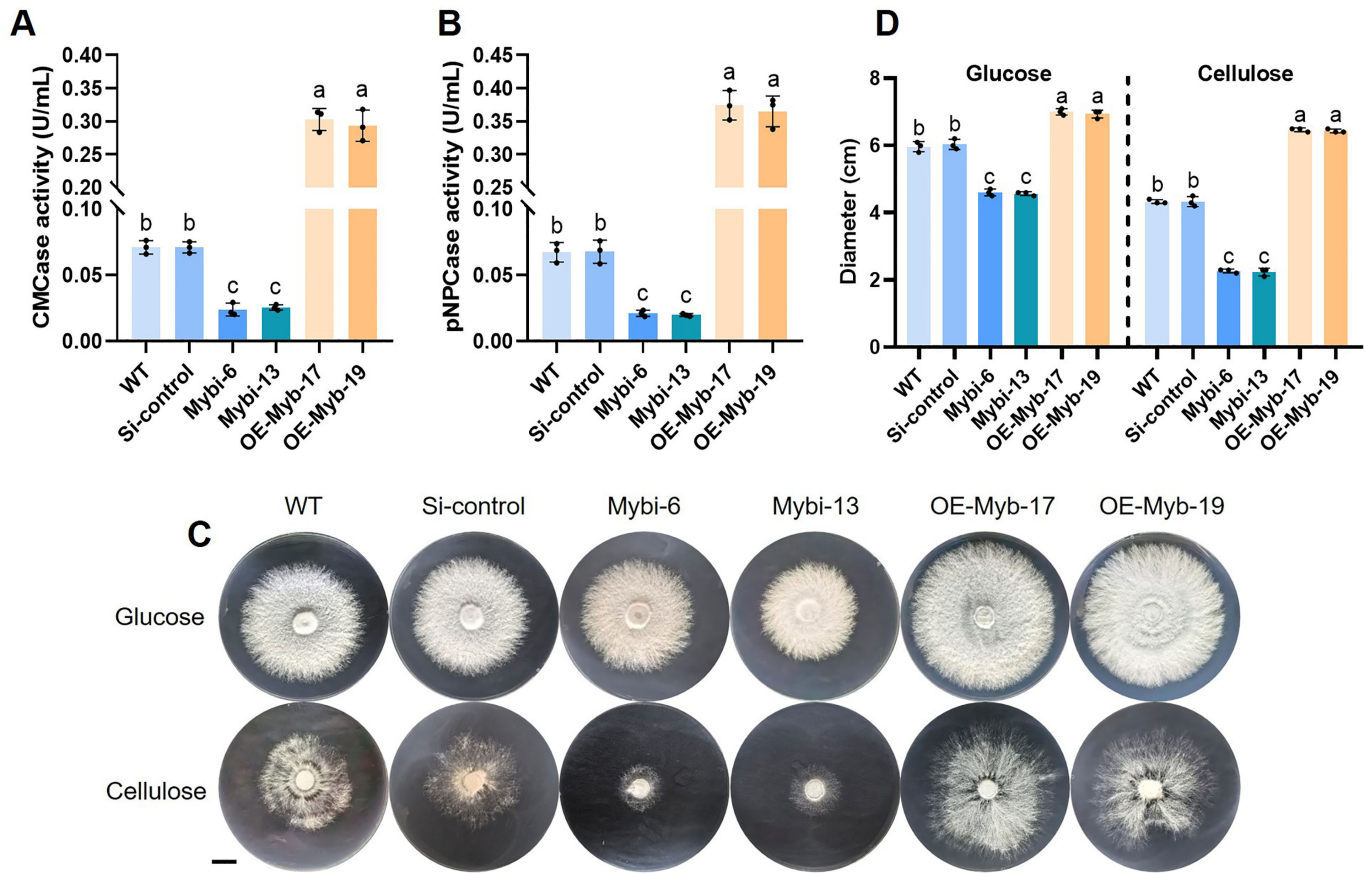
*Gl*Myb contributes to cellulase activity and cellulose utilization. The endocellulase (**A**) and exoglucanase (**B**) activities were determined in the WT, Si-control, *GlMyb*-silenced, and *GlMyb*-overexpressing strains. (**C and D**) The growth phenotypes and hyphal diameter of the WT, Si-control, *GlMyb*-silenced, and *GlMyb*-overexpressing strains were assessed following 7 days of cultivation on glucose or sodium microcrystalline cellulose (CMC-Na) medium (scale bar = 1 cm). Data are presented as the mean ± SD (*n* = 3). Statistical significance is represented by different letters corresponding to *P* < 0.05 based on Tukey’s multiple range test.

To elucidate the regulatory mechanism of *Gl*Myb on cellulose utilization, cellulase activity was detected in the WT, Si-control, *GlMyb*-silenced, and *GlMyb*-overexpressing strains, which were established in our previous study (42). The results showed that the endocellulase activity was reduced by 67% in the *GlMyb*-silenced strains and increased by 328% in the *GlMyb*-overexpressing strains, compared with the WT strains (Fig. 5A). Similarly, the exoglucanase activity in the *GlMyb*-silenced strains (70%) was significantly less than that of the WT and Si-control strains, while it was significantly increased by 452% in the *GlMyb*-overexpressing strains (Fig. 5B). Furthermore, we cultured these strains for 7 days on medium containing either glucose or CMC-Na as the sole carbon source and assessed their growth phenotypes (Fig. 5C). As illustrated in Fig. 5D, in the presence of glucose, the mycelial diameter of the *GlMyb*-silenced strains decreased by 23% compared to the control strains, while the *GlMyb*-overexpressing strains increased by 16%. In the presence of cellulose, the *GlMyb*-silenced strains exhibited a 50% reduction in hyphal diameter compared to the control strains, whereas the *GlMyb*-overexpressing strains showed a 34% increase. These results indicate that *Gl*Myb participates in hyphal growth under both glucose and cellulose conditions. However, in cellulose-containing growth medium, the degree of inhibition or enhancement in *GlMyb*-silenced or *GlMyb*-overexpressing strains is significantly higher compared to that in non-cellulose growth medium.

**Fig 6:**
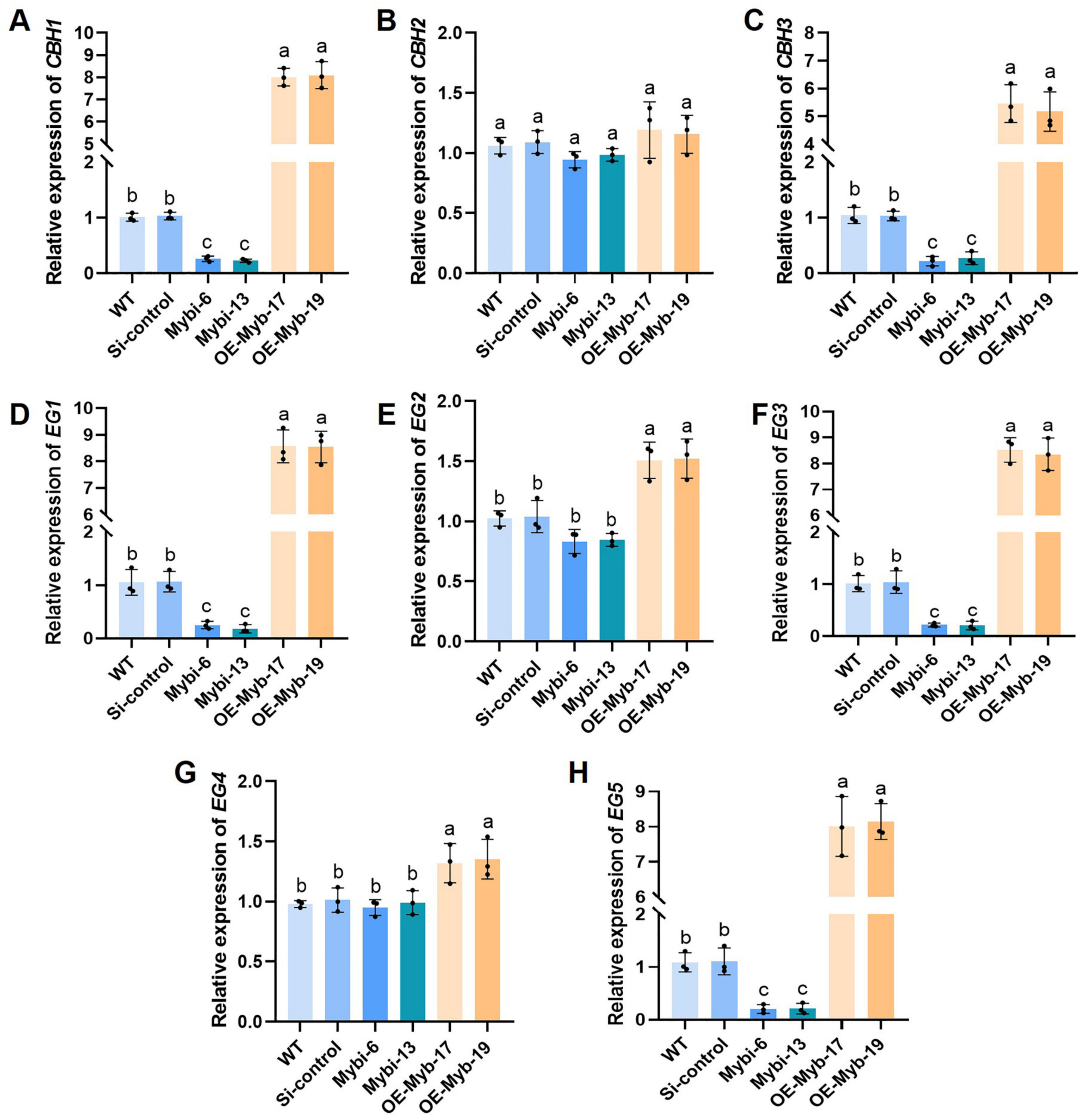
The relative expression levels of different *CBHs* and *EGs* genes. The relative expression levels of *CBH1* (**A**), *CBH2* (**B**), *CBH3* (**C**), *EG1* (**D**), *EG2* (**E**), *EG3* (**F**), *EG4* (**G**), and *EG5* (**H**) in the WT, Si-control, *GlMyb*-silenced, and *GlMyb*-overexpressing strains. Data are presented as the mean ± SD (*n* = 3). Statistical significance is represented by different letters corresponding to *P* < 0.05 based on Tukey’s multiple range test.

To study the potential *Gl*Myb’s target genes that are involved in cellulose utilization, the *CBHs* and *EGs* genes expression was examined. The expression of the *CBH1* and *CBH3* genes was significantly reduced in the *GlMyb*-silenced strains compared with that in the control strains (Fig. 6A and C). By contrast, the expression of these two genes was strongly increased in the *GlMyb*-overexpressing strains. Crucially, the expression of *CBH2* showed no significant variation among these strains (Fig. 6B), which was similar to that in the *GlSlt2*-silenced and *GlSlt2*-overexpressing strains (Fig. 2B). As well, the expression of *EG1*, *EG3,* and *EG5* genes was evidently downregulated in the *GlMyb*-silenced strains and obviously upregulated in the *GlMyb*-overexpressing strains, whereas that of *EG2* and *EG4* genes did not show any significant variation across all the strains (Fig. 6D through H). Together, these findings suggest that *Gl*Myb plays an active role in the cellulose utilization of *G. lucidum* by modulating the expression of cellulase-related genes.

### *Gl*Myb directly binds to the promoters of *CBHs* and *EGs* genes

**Fig 7:**
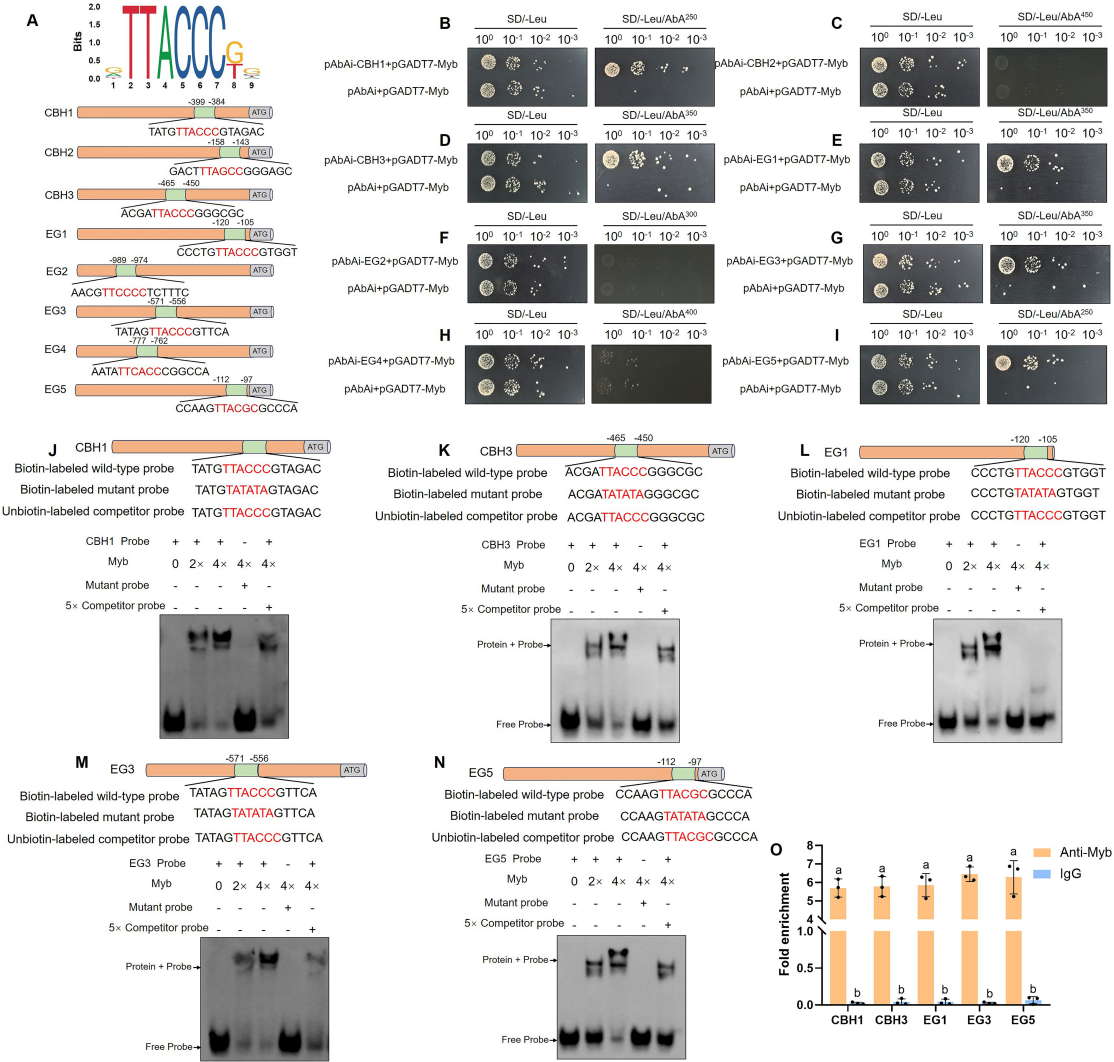
Direct binding of *Gl*Myb to the promoters of *CBHs* and *EGs* genes. (**A**) *Gl*Myb binding motif predicted by JASPAR software. Y1H assay verification between *Gl*Myb and *CBH1* (**B**), *CBH2* (**C**), *CBH3* (**D**), *EG1* (**E**), *EG2* (**F**), *EG3* (**G**), *EG4* (**H**), and *EG5* (**I**) promoters. EMSA assays show that the His-Myb fusion protein binds to the promoters of *CBH1* (**J**), *CBH3* (**K**), *EG1* (**L**), *EG3* (**M**), and *EG5* (**N**). Biotin-labeled binding motifs predicted by JASPAR were used as wild-type probes. Mutant probes were generated by substituting the core motif with “TATATA” followed by biotin labeling. Unlabeled wild-type probes served as competitors in binding assays. – or + represents absence or presence, respectively. (**O**) ChIP-qPCR analysis of *Gl*Myb binding to cellulase-related gene promoters in WT collected from the liquid culture medium with microcrystalline cellulose as the sole carbon source. Immunoprecipitation was performed using anti-*Gl*Myb antibody, with IgG serving as a negative control. Immunoprecipitate (IP)/input was calculated by comparison with the threshold cycle (C_T_) values between the immunoprecipitate and input. Data are presented as the mean ± SD (*n* = 3). Statistical significance is represented by different letters corresponding to *P* < 0.05 based on Tukey’s multiple range test.

To elucidate the mechanism by which *Gl*Myb modulates the expression of *CBHs* and *EGs* genes, the direct regulatory effect of *Gl*Myb on these genes was investigated. Previous studies suggested that the binding element of *Gl*Myb was deduced to be [A/G] TTAC [G/C] [C/G] in *G. lucidum* (42). To verify the above speculation, the JASPAR database (https://jaspar.elixir.no/) and inferred binding motifs were used to analyze the *Gl*Myb-binding sites with the promoters of *CBHs* and *EGs* genes (Fig. 7A), and a possible binding site with the highest score was selected for verification (the higher the score, the higher the credibility). Thereafter, a yeast one-hybrid (Y1H) assay was performed. To amplify the signal intensity, each predicted binding motif (16 bp) was tandemly repeated in triplicate and subsequently cloned into the pAbAi vector. As shown in Fig. 7B through I, Y1HGold yeast strains cotransformed with pGADT7-Myb and pAbAi-CBH1/CBH3/EG1/EG3/EG5 grew well in a synthetically defined medium lacking Leu (SD/-Leu) with different concentrations of aureobasidin A (AbA), while those containing pGADT7-Myb and pAbAi-CBH2/EG2/EG4 did not grow. These results confirm that the promoters of *CBHs* and *EGs* genes bound to the transcription factor *Gl*Myb of *G. lucidum*.

To further validate the binding of *Gl*Myb with these promoters, electrophoretic mobility shift assay (EMSA) was conducted (Fig. 7J through N). As expected, *Gl*Myb bound to the regulatory regions of *CBH1*, *CBH3*, *EG1*, *EG3,* and *EG5* genes by recognizing the predicted binding motif, and as the amount of *Gl*Myb protein increased, the intensity of the mobility shift signal gradually increased (Fig. 7J through N, *lanes 1–3*). Furthermore, it is worth noting that with the same concentration of mutated probes, the mobility shift was not clearly observed (Fig. 7J through N, *lane 4*). In competition assays, with an excess of competitor probe, the intensity of mobility shift decreased (Fig. 7J through N, *lane 5*), indicating that these bindings were specific. To determine whether *Gl*Myb directly associates with the promoters of these cellulase-related genes *in vivo*, chromatin immunoprecipitation quantitative PCR (ChIP-qPCR) assays were performed using anti-*Gl*Myb antibody. The predicted ciselement-containing promoter regions of *CBH1*, *CBH3*, *EG1*, *EG3,* and *EG5* genes were enriched by anti-*Gl*Myb antibody compared with IgG control (Fig. 7O). Therefore, these results confirmed that *Gl*Myb directly binds to the predicted binding motifs in the promoter of *CBHs* and *EGs* genes.

### The S245 site of *Gl*Myb is a key factor affecting its binding ability to the promoter of target genes

**Fig 8:**
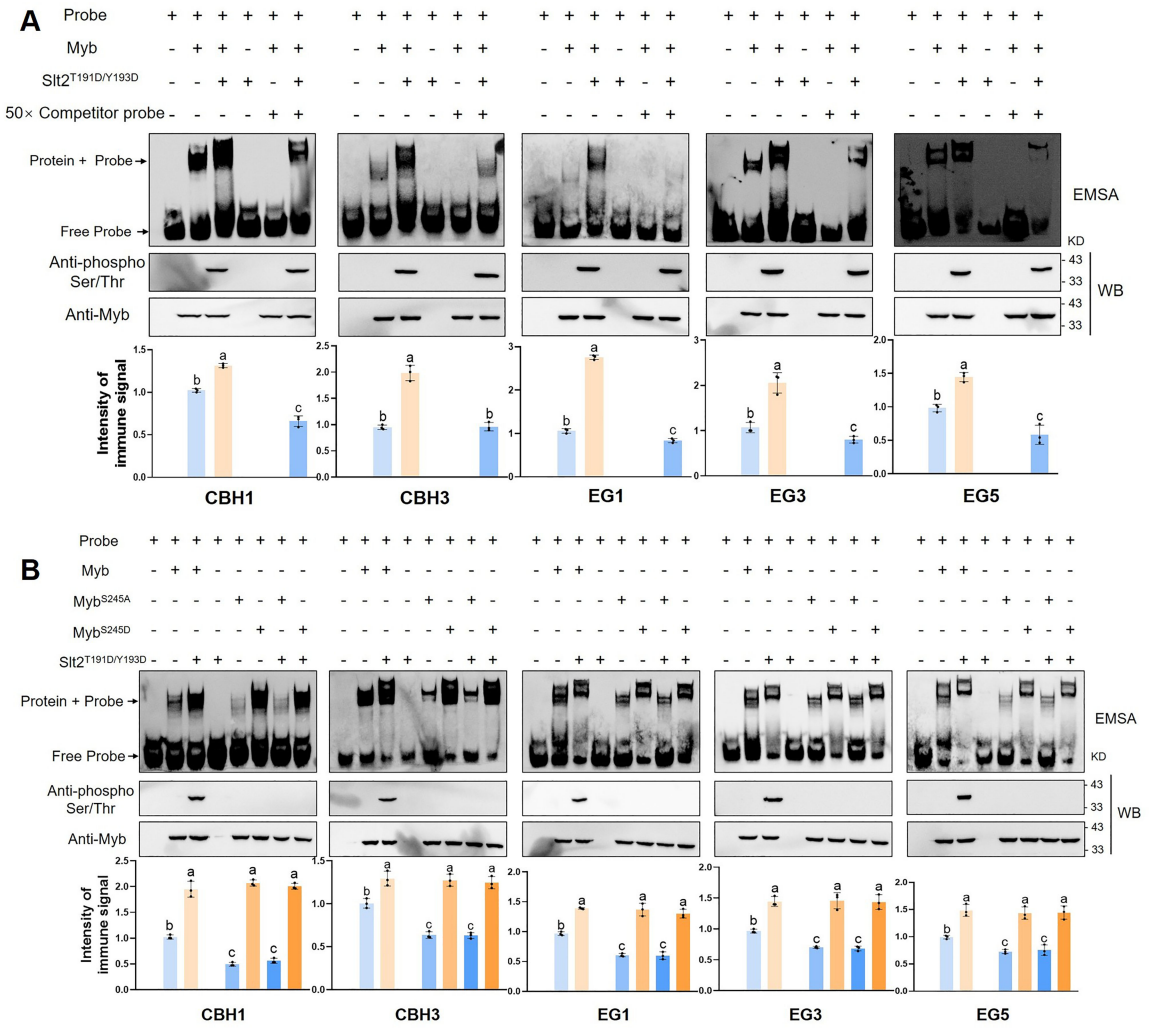
Effect of *Gl*Slt2 on the binding ability of *Gl*Myb to the promoters of *CBHs* and *EGs* genes. (**A**) EMSA showing the binding of *Gl*Myb to the promoter of *CBH1*, *CBH3*, *EG1*, *EG3,* and *EG5* genes after incubation with purified His-Slt2^T191D/Y193D^ fusion protein in protein kinase buffer at 30°C for 30 min. Subsequently, the mixture was incubated with biotin-labeled wild-type probes in the presence or absence of unlabeled competitor probes at 25°C for 20 min. (**B**) EMSA showing the S245 site of *Gl*Myb affects its binding to the promoters of the *CBH1*, *CBH3*, *EG1*, *EG3,* and *EG5* genes. The His-Myb, His-Myb^S245A^, and His-Myb^S245D^ proteins were incubated with His-Slt2^T191D/Y193D^ in kinase assay buffer at 30°C for 30 min. After reactions, the mixtures, as well as His-Myb, His-Myb^S245A^, His-Myb^S245D^, or His-Slt2^T191D/Y193D^ protein, were added with biotin-labeled probes for EMSA assays. – or + represents absence or presence, respectively. The Image J software was used to quantify the gel band intensities. The value in the control (His-Myb alone) was set as 1. Data are presented as the mean ± SD (*n* = 3). Statistical significance is represented by different letters corresponding to *P* < 0.05 based on Tukey’s multiple range test.

To determine whether *Gl*Slt2 affects *Gl*Myb-mediated transcriptional activity, the binding of phosphorylated *Gl*Myb to the target gene promoter was investigated using EMSA assays (Fig. 8A). Compared to *Gl*Myb alone (Fig. 8A, *lane 2*), the binding strength of *Gl*Myb to the target promoter was enhanced by adding purified *Gl*Slt2^T191D/Y193D^ to the reaction (Fig. 8A, *lane 3*). However, in the presence of *Gl*Slt2^T191D/Y193D^ alone (Fig. 8A, *lane 4*), no DNA-binding capacity was detectable, which was similar to that of probes only (Fig. 8A, *lane 1*). Upon the addition of a competitor probe to the reaction system, the binding complex was completely abolished in samples containing *Gl*Myb alone (Fig. 8A, *lane 5*). By contrast, the binding complex remained detectable when both *Gl*Slt2^T191D/Y193D^ and *Gl*Myb were present (Fig. 8A, *lane 6*). To further elucidate the functional significance of the S245 site in *Gl*Myb’s promoter-binding activity, we generated and purified the phosphomimetic *Gl*Myb^S245D^ recombinant protein, which was subsequently subjected to EMSA assay (Fig. 8B). Compared to *Gl*Myb alone (Fig. 8B, *lane 2*), *Gl*Myb^S245A^ exhibits reduced binding strength irrespective of *Gl*Slt2 presence (Fig. 8B, *lanes 5, 7*), whereas *Gl*Myb^S245D^ enhances binding strength (Fig. 8B, *lanes 6, 8*), consistent with the scenario where *Gl*Myb and *Gl*Slt2 coexist (Fig. 8B, *lane 3*). Overall, these results establish that the S245 site of *Gl*Myb is a key factor affecting its binding ability to the promoter of target genes.

### *Gl*Slt2 positively regulates *Gl*Myb-mediated cellulose utilization

**Fig 9:**
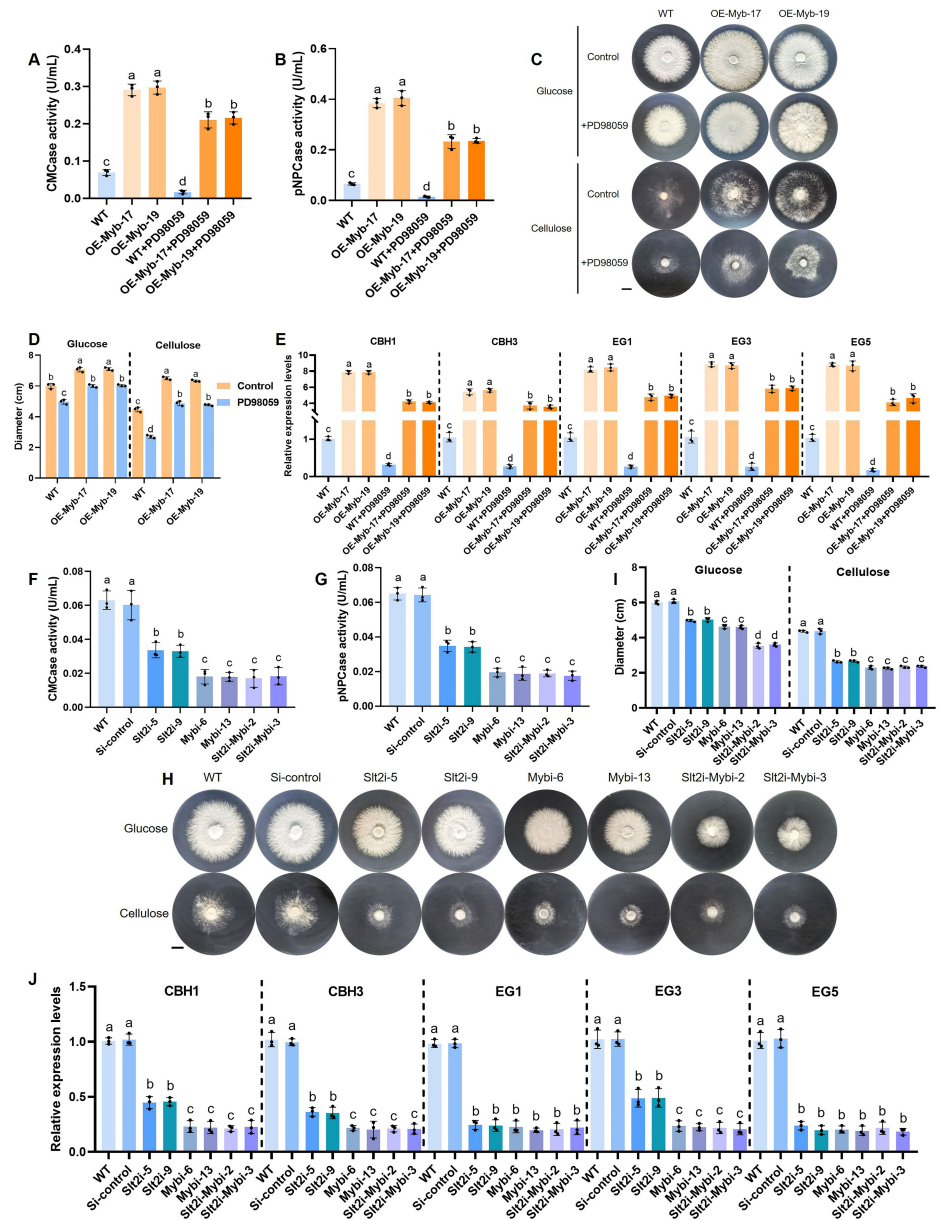
Detection of cellulase activity and cellulose utilization growth phenotypes, as well as cellulase-related genes transcript levels. The endocellulase (**A**) and exoglucanase (**B**) activities were determined in the WT and *GlMyb*-overexpressing strains with or without PD98059 treatment. (**C and D**) The growth phenotypes and hyphal diameter of the WT and *GlMyb*-overexpressing strains with or without PD98059 treatment were assessed following 7 days of cultivation on glucose or sodium microcrystalline cellulose (CMC-Na) medium (scale bar = 1 cm). (**E**) The relative expression levels of *CBH1*, *CBH3*, *EG1*, *EG3,* and *EG5* in the WT and *GlMyb*-overexpressing strains with or without PD98059 treatment. The endocellulase (**F**) and exoglucanase (**G**) activities were determined in the WT, Si-control, *GlSlt2*-silenced, *GlMyb*-silenced, and *GlSlt2-GlMyb* double-silenced strains. (**H and I**) The growth phenotypes and hyphal diameter of the WT, Si-control, *GlSlt2*-, *GlMyb*-, and *GlSlt2-GlMyb* double-silenced strains were assessed following 7 days cultivation on glucose or sodium microcrystalline cellulose (CMC-Na) medium (scale bar = 1 cm). (**E**) The relative expression levels of *CBH1*, *CBH3*, *EG1*, *EG3,* and *EG5* in the WT and *GlMyb*-overexpressing strains. Data are presented as the mean ± SD (*n* = 3). Statistical significance is represented by different letters corresponding to *P* < 0.05 based on Tukey’s multiple range test.

To explore the genetic interactions between *Gl*Slt2 and *Gl*Myb, the specific phosphorylation inhibitor (PD98059) of *Gl*Slt2 was added to the *GlMyb*-overexpressing strains. Subsequently, cellulase activity was examined under PD98059 treatment. We noticed that the endocellulase activity in the *GlMyb*-overexpressing strains was much stronger than that in the WT strain (Fig. 9A). After treatment with PD98059 in the WT strain, the endocellulase activity in the treated WT strain strongly decreased (75%) compared with the untreated WT strain. Notably, the endocellulase activity in the *GlMyb*-overexpressing strains after treatment with PD98059 was clearly higher (11.3-fold) than that of the treated WT strain, but lower (28%) than that of the untreated *GlMyb*-overexpressing strains. Consistently, the exoglucanase activity was manifestly increased in the *GlMyb*-overexpressing strains relative to the WT strain (Fig. 9B). Post-treatment analysis revealed a marked reduction in the activity of the WT strain, exhibiting a substantial 77% decrease. Conversely, the treated *GlMyb*-overexpressing strains demonstrated relatively elevated activity (14.7-fold) compared to the treated WT strain, yet it was observed to be diminished (41%) in comparison to the untreated *GlMyb*-overexpressing strains. These data elucidate that *Gl*Slt2 is involved in *Gl*Myb-mediated cellulase activity. Equally, mycelial growth phenotypes were evaluated in medium supplemented with glucose or CMC-Na as the sole carbon source, with or without PD98059 treatment (Fig. 9C). The results showed that in the presence of glucose, the mycelial diameter of the *GlMyb*-overexpressing strains was significantly larger than that of the WT strain (Fig. 9D). Treatment with PD98059 significantly inhibited growth in both the WT (16% inhibition) and *GlMyb*-overexpressing (14% inhibition) strains compared to untreated controls. In the presence of cellulose, upon PD98059 treatment, the WT and *GlMyb*-overexpressing strains displayed 40% and 26% growth inhibition, respectively, relative to untreated controls (Fig. 9D). These results indicate that the growth inhibition of PD98059-treated *GlMyb*-overexpressing strains is significantly higher in the presence of cellulose than in non-cellulose medium. Moreover, we assessed the expression of *CBHs* and *EGs* genes after PD98059 treatment. Surprisingly, in the *GlMyb*-overexpressing strains, these genes (*CBH1*, *CBH3*, *EG1*, *EG3,* and *EG5*) were robustly upregulated relative to the untreated WT strain, while they were dramatically downregulated, with a reduction in expression levels by approximately 70%–85% in the treated WT strain (Fig. 9E). More significantly, the expression levels of these genes were distinctly reduced in the *GlMyb*-overexpressing strains following treatment, exhibiting a decrease of approximately 20%–50% compared to the untreated *GlMyb*-overexpressing strains. Nonetheless, their expression levels are considerably elevated in comparison to the treated WT strain, with an upregulation of approximately threefold to fivefold. To investigate the effect of *Gl*Slt2 on *Gl*Myb’s regulation of cellulase-related gene expression *in vivo*, WT and *Gl*Myb-overexpressing strains were cultured in a medium with CMC-Na as the sole carbon source, with or without the addition of PD98059, and ChIP-qPCR was performed using *Gl*Myb antibody (Fig. S5). The result indicates that *Gl*Myb-overexpressing strains exhibited sixfold to sevenfold higher fold enrichment of the five cellulase genes compared to the WT strain. Following PD98059 treatment, the WT strain exhibited a 50%–80% reduction in fold enrichment. While PD98059 treatment led to a 40%–50% decrease in fold enrichment in *Gl*Myb-overexpressing strains relative to untreated strains, these values remained significantly higher than those observed in treated WT strains.

To prevent the off-target effects caused by the addition of PD98059, we employed RNAi technology to screen for double-silenced strains of *GlSlt2-GlMyb* (Slt2i-Mybi-2 and Slt2i-Mybi-3) and examined the transcriptional and protein levels of the candidate strains to ensure their effectiveness (Fig. S6A through C). Subsequently, the endocellulase and exoglucanase activities were measured in the controls, *GlSlt2*-silenced, *GlMyb*-silenced, and *GlSlt2-GlMyb* double-silenced strains. The enzymatic assays demonstrated that the *GlSlt2-GlMyb* double-silenced strains exhibited significantly reduced endocellulase and exoglucanase activities compared to the *GlSlt2*-silenced strains, with levels comparable to those observed in the *GlMyb*-silenced strains (Fig. 9F and G). In addition, these strains were cultured on medium with glucose and CMC-Na as the sole carbon sources, and their growth phenotypes were evaluated (Fig. 9H). The results showed that in the presence of glucose, significant growth inhibition was observed in both the *GlSlt2*-silenced (16%) and *GlMyb*-silenced (23%) strains compared to the WT. Notably, the *GlSlt2-GlMyb* double-silenced strains exhibited even stronger inhibition (40%) (Fig. 9I, *left*). In the presence of cellulose, the *GlSlt2-GlMyb* double-silenced strains showed inhibition (50%) comparable to the *GlMyb*-silenced strains but more severe than the *GlSlt2*-silenced strains (Fig. 9I, *right*), implying that in the presence of glucose, *Gl*Slt2 may function through pathways other than *Gl*Myb, whereas in the presence of cellulose, *Gl*Slt2 regulates cellulose utilization by affecting *Gl*Myb phosphorylation. Then, the transcriptional levels of these five genes (*CBH1*, *CBH3*, *EG1*, *EG3,* and *EG5*) were analyzed, and ChIP-qPCR using an anti-*Gl*Myb antibody was performed in these strains, and the results showed that compared with the *GlSlt2*-silenced strains, both the expression and the fold enrichment of *CBH1*, *CBH3*, and *EG3* were significantly reduced in the *GlSlt2-GlMyb* double-silenced strains, similar to the *GlMyb*-silenced strains (Fig. 9J; Fig. S7). By contrast, neither the expression levels nor the *Gl*Myb-binding enrichment of *EG1* and *EG5* exhibited significant differences among *GlSlt2-*, *GlMyb-*, and *GlSlt2-GlMyb* double-silenced strains. In summary, these results indicate that *Gl*Slt2 positively regulates *Gl*Myb-mediated cellulose utilization.

## DISCUSSION

Cellulose-rich biomass, including agricultural and forestry by-products, such as straw, corn cobs, cottonseed hulls, and sawdust, represents a significant resource for the cultivation of edible fungi (4, 11). The utilization of these by-products not only enhances resource efficiency but also provides a sustainable substrate for fungal cultivation. Edible fungi, through the action of cellulose-degrading enzymes secreted by their mycelium, break down cellulose and convert carbon sources and nutrients into valuable fruiting bodies. Furthermore, the efficient utilization of cellulose from agricultural and forestry by-products can lead to its transformation into valuable reusable resources, including fertilizers. This process exemplifies the conversion of agricultural and forestry waste into a high-value product through the agency of fungal biotransformation (10). The mitogen-activated protein kinase (MAPK) pathway is a crucial and evolutionarily conserved signal transduction pathway in all eukaryotes. Studies have reported that the MAPK kinases Tmk2 and Tmk3 are involved in cellulase production in *Trichoderma reesei* (43, 44). In addition, the hyperosmotic response (OS) MAP kinase pathway is involved in the cellulolytic response in *Neurospora crassa* (45). In our research, we demonstrated that MAPK kinase *Gl*Slt2 plays a positive role in regulating cellulase activity and cellulose utilization in *Ganoderma lucidum*. Furthermore, *Gl*Slt2 was found to significantly modulate the expression of cellulase-related genes. Interestingly, our results contrast with previous findings regarding the deletion of the Slt2 homolog *Tmk2* in *T. reesei*, which led to an increase in cellulolytic activity (26). This discrepancy may stem from the functional diversification of orthologous proteins across different species, highlighting the complexity of conserved pathways in various biological contexts.

As a protein kinase, Slt2 primarily engages in processes such as growth, development, and cell wall integrity through the mediation of phosphorylation (41, 46). Previous investigations have identified Slt2-interacting proteins, including transcription factors Rlm1 and Swi4, the cyclin C Ssn8, the RNA polymerase II (Pol II) catalytic subunit Rpb1, and the phosphoprotein Sir3 (47–49). Nevertheless, numerous downstream target proteins of Slt2 remain to be identified. In our study, a Y2H screening library was utilized to identify 13 potential *Gl*Slt2-binding proteins. Notably, among these candidates, no cellulase-related genes were identified. However, a MYB-type transcription factor, *Gl*Myb, which may interact with *Gl*Slt2, was discovered. As evidenced by the Y2H, BiFC, and Co-IP assays, *Gl*Slt2 interacted with *Gl*Myb both *in vitro* and *in vivo*. In prior research, Myb transcription factors have been implicated in regulating the expression of cellulase-related genes. For instance, in *Arabidopsis*, MYB46 is involved in the synthesis of cellulose in secondary cell walls by transcriptionally activating the cellulose synthase genes of *CESA4*, *CESA7*, and *CESA8* (50). In addition, the MYB motif present in the promoter region of the cellulase gene *CelB* in *Dictyostelium discoideum* suggests a potential binding site for MYB proteins, thereby modulating the expression of the *CelB* gene (51). Consistent with our results, *Gl*Myb was found to positively modulate the expression of cellulase-related genes, including *CBH1*, *CBH3*, *EG1*, *EG3*, and *EG5*, in *G. lucidum*. However, the expression of *CBH2*, *EG2*, and *EG4* appears to be unaffected by *Gl*Myb regulation. To predict the functional diversity of *Gl*Myb, the distribution of *Gl*Myb binding motifs in the *G. lucidum* genome was analyzed through KEGG analysis, revealing that *Gl*Myb-binding sequences are distributed in pathways such as “MAPK signaling pathway,” “Carbon metabolism,” “Amino acid metabolism,” “Protein folding,” “Cellular transport pathways,” and “Lignocellulose degradation” (Fig. S8). The findings indicate that *Gl*MYB in *G. lucidum*, in addition to affecting cellulose utilization, possesses a wide range of functions, and the related regulatory mechanisms require further investigation.

MAP kinases typically modulate cellular processes by phosphorylating downstream proteins or affecting their activity (21, 52). For example, the MAPK protein kinase Slt2-mediated phosphorylation of Mrc1 following heat shock leads to a delay in DNA replication and promotes extensive transcriptional reprogramming. Mrc1, an evolutionarily conserved factor associated with the replisome, is crucial for efficient DNA replication (53). Slt2-mediated phosphorylation of Rlm1 is essential for activating its transcriptional activity (54). In our study, we discovered that in the presence of cellulose, *Gl*Slt2 phosphorylates the S245 site of *Gl*Myb. The S245 site of *Gl*Myb is a key factor affecting its binding ability to the promoter of cellulase-related genes (*CBH1*, *CBH3*, *EG1*, *EG3*, and *EG5*). It is well known that Slt2 is involved in the cell wall integrity pathway, and it has been previously reported that Slt2 regulates fungal cellulose utilization. However, whether the two pathways are related remains unknown. Notably, in *T. reesei*, the Slt2 homolog Tmk2 has been shown to suppress cellulase production by maintaining cell wall integrity (43). In our study, in the presence of glucose or under cell wall stress, *Gl*Slt2 has no significant effect on the phosphorylation of *Gl*Myb. While in the presence of cellulose, *Gl*Slt2 phosphorylates *Gl*Myb. These findings demonstrate that *Gl*Slt2 is involved in the cell wall integrity pathway, but not through the phosphorylation of *Gl*Myb. The phenotype of cellulose utilization mediated by *Gl*Slt2 phosphorylation of *Gl*Myb is independent of the classical cell wall integrity pathway in *G. lucidum*, indicating a different mechanism from that in *T. reesei*. In addition, *Gl*Myb has been reported to regulate non-cellulose-related phenotypes in *G. lucidum*. For instance, it was found that *Gl*Myb directly binds to the promoters of spermidine synthase (spds1 and spds2) in *G. lucidum* under heat stress (42), indicating that *Gl*Myb is involved in heat stress and cellulose utilization.

**Fig 10:**
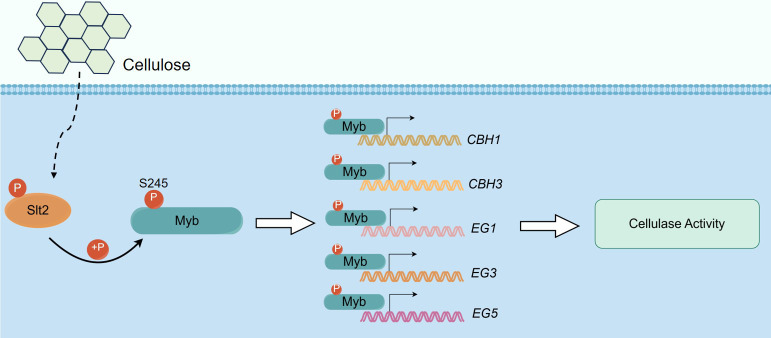
The working model of *Gl*Slt2 participated in *Gl*Myb regulation of cellulase activity and cellulose utilization. In the presence of cellulose, *Gl*Slt2 phosphorylates the S245 site of *Gl*Myb and promotes the binding of *Gl*Myb to the promoters of cellulase-related genes (*CBH1*, *CBH3*, *EG1*, *EG3*, and *EG5*), resulting in increased cellulase activity.

Collectively, our findings demonstrate the identification of *Gl*Myb as an interaction partner of *Gl*Slt2, with their interaction confirmed through both *in vivo* and *in vitro* assays. Our research further elucidates that *Gl*Slt2 phosphorylates the S245 site of *Gl*Myb and positively regulates cellulose utilization (Fig. 10). These data not only establish a framework for the regulation of cellulose utilization by *Gl*Slt2 in macrofungi but also lay the foundation for improvements in biomass conversion and carbon source utilization.

## MATERIALS AND METHODS

### Strains and growth conditions

The wild-type (WT) strain of *G. lucidum* (ACCC53264) was sourced from the Agricultural Culture Collection of China. The empty RNAi-vector control strain (Si-control), *GlSlt2*-silenced strains (Slt2i-5 and Slt2i-9), *GlMyb*-silenced strains (Mybi-6 and Mybi-13), and *GlMyb*-overexpressing strains (OE-Myb-17 and OE-Myb-19) were established in our previous study (37, 42).

To evaluate the utilization of cellulose, different strains were cultured in the dark at 28°C for 7 days on medium with glucose or sodium carboxymethyl cellulose (CMC-Na) as the sole carbon source, followed by photography. The medium with glucose as the sole carbon source contained (wt/vol): 2% glucose, 0.46% KH_2_PO_4_, 0.05% MgSO_4_·7H_2_O, 0.5% (NH_4_)_2_SO_4_, and 2 mL/L trace elements (0.5% MnSO_4_·4H_2_O, 0.85% H_3_BO_3_, 0.03% (NH_4_)_6_Mo_7_O_24_·4H_2_O, 0.6% MnSO_4_, 0.06% CuSO_4_·5H_2_O, 0.27% ZnSO_4_·7H_2_O). The medium with CMC-Na as the sole carbon source contained (wt/vol): 1% CMC-Na, 0.46% KH_2_PO_4_, 0.05% MgSO_4_·7H_2_O, 0.5% (NH_4_)_2_SO_4_, and 2 mL/L trace elements. To assess enzyme activities and the transcriptional level of cellulase-related genes, all strains were initially cultivated in a liquid medium with glucose as the sole carbon source at 28°C and 150 rpm for 5 days, then transferred the same biomass of different strains to a liquid medium with microcrystalline cellulose as the sole carbon source (containing 1% microcrystalline cellulose, 0.46% KH_2_PO_4_, 0.05% MgSO_4_·7H_2_O, 0.5% (NH_4_)_2_SO_4_, and 2 mL/L trace elements) and incubated at 28°C and 150 rpm for an additional 2 days (36). For cell wall stress, add 1 mg/mL calcofluor white (CFW) or 4 mg/mL congo red (CR) to the medium with glucose as the sole carbon source.

### Plasmid construction

The RNAi vector plasmid pAN7-ura3-dual and the pGPiE plasmid were previously constructed by our laboratory for obtaining silenced strains and overexpressing strains, respectively (36, 55). For the construction of the silenced vector, *G. lucidum* cDNA was used as a template to amplify the *GlSlt2* gene and *GlMyb* gene fragments. Then, *Gl*Slt2i-F/R and *Gl*Mybi-F/R primers (listed in Table S2) were used for joint PCR to clone into the RNAi vector. For the construction of the overexpressing vector, the full-length coding sequence (CDS) of the *GlSlt2* gene was amplified with the OE-Slt2-F/R (listed in Table S2) and cloned into the pGPiE overexpression vector.

The yeast two-hybrid vectors pGADT7 and pGBKT7 were purchased from Clontech (Mountain View, CA, USA). The full-length *GlSlt2* gene was inserted into the pGBKT7 bait vector (pGBKT7-Slt2), and the full-length *GlMyb* gene and truncated versions (*GlMyb*^1-163^, *GlMyb*^164-629^) were individually cloned into the pGADT7 prey vector (pGADT7-Myb, pGADT7-Myb^1-163^, and pGADT7-Myb^164-629^).

The bimolecular fluorescent plasmids pVN1 and pVC1 were constructed according to the methods described previously (56). Plasmid pVN1 (CEN URA3 P_MET25_-Venus-N-T_CYC1_) was generated by replacing the *yEGFP3* gene (an *Xba*I-*Bam*HI fragment) in pUG36 with the *Xba*I-*Bam*HI fragment of Venus-N encoding aa 1–173 of Venus, a GFP variant. Similarly, pVC1 (CEN HIS3 P_MET25_-Venus-C-T_CYC1_) was generated by replacing the *yEGFP3* gene in pUG34 with the *Xba*I-*Bam*HI fragment of Venus-C encoding aa 155–238 of Venus. A linker encoding four alanines was added after Venus-N and Venus-C. Venus-N and Venus-C genes were amplified from plasmids kindly provided by Won-Ki Huh (57). The full-length *GlSlt2* gene was inserted into the pVN1 (PVN-Slt2), carried with the fragment of Venus-N. The full-length *GlMyb* gene and truncated versions (*GlMyb*^1–163^, *GlMyb*^164–629^) were individually cloned into the pVC1 (PVC-Myb, PVC-Myb^1–163^, and PVC-Myb^164–629^), carried with the fragment of Venus-C.

The protein expression vector used was pColdI (TaKaRa, Shiga, Japan). The full-length *GlSlt2, GlMyb* gene, and mutant versions were inserted into the pColdI (pColdI-Slt2, pColdI-Slt2^T191D/Y193D^, pColdI-Myb, pColdI-Myb^S245A^, and pColdI-Myb^S245D^).

The yeast one-hybrid (Y1H) assay used the pAbAi plasmid (Invitrogen, Carlsbad, CA, USA). As predicted by the JASPAR software, the promoter regions of *CBHs* and *EGs* genes were tandemly repeated three times according to the previously described method (58), and subsequently cloned into the pAbAi vector to generate the recombinant construct (pAbAi-*CBH1*, *CBH2*, *CBH3*, *EG1*, *EG2*, *EG3*, *EG4*, *EG5*). The primers listed in Table S2.

### Construction of RNAi and overexpressing strains

*GlSlt2-GlMyb* double-silenced strains and *GlSlt2*-overexpressing strains were constructed and screened as previously described (42, 59). Briefly, the RNAi silencing vector pAN7-dual-Slt2i-Mybi was electroporated into *G. lucidum*. The constructed pGPiE-Slt2 plasmid was transformed into *G. lucidum* using *Agrobacterium tumefaciens*-mediated transformation. Subsequently, the two independent strains with the highest silenced or overexpressing efficiency were selected for subsequent experiments.

### Gene expression analysis

Quantitative RT-qPCR analysis was conducted using cDNA from the silenced or overexpressing candidate strains and primers detailed in Table S2, with the Eppendorf Mastercycler Ep Realplex 2.2 software. Gene expression levels were determined relative to 18S RNA, employing the 2^−ΔΔCT^ method for normalization, consistent with our previous determination of reliable reference genes for expression studies.

### Enzyme activity detection

Endocellulase activity (CMCase), exoglucanase activity (pNPCase), and β-glucosidase activity were assessed using established protocols (9, 60, 61). In brief, the culture supernatants from liquid culture medium were used for CMCase analysis with a DNS reagent against 1% CMC-Na; the absorbance at 540 nm was measured after a 30 min incubation at 50°C. For pNPCase assays, the culture supernatants were collected and mixed with p-nitrophenol-D-cellobioside (pNPC) in citrate phosphate buffer (CPB, 0.2 M, pH 6.0) for 10 min at 65°C. The amount of *p*-nitrophenol (*p*NP) released was measured at a wavelength of 405 nm. β-glucosidase activity was determined by monitoring *p*NP release from pNPG. 100 µL of secretome was incubated with 100 µL of 1 mM pNPG for 30 min, and the reaction was halted with 1 M sodium carbonate (pH 11.5), with *p*NP quantified at 410 nm against a standard curve.

### Co-IP

Co-immunoprecipitation (Co-IP) assays were conducted following established methods (59). The WT strain (0.15 g), collected from the liquid culture medium with microcrystalline cellulose as the sole carbon source, was lysed in a buffer with 100 mM NaCl, 20 mM Tris–HCl at pH 7.6, 0.1% Triton X-100, a protease inhibitor cocktail, and 1 mM phenylmethanesulfonyl fluoride for 1 h. The supernatant (200 µL) was incubated with protein A/G agarose (Thermo Fisher Scientific, Illkirch-Graffenstaden, France) and either control rabbit IgG or anti-*Gl*Slt2 antibody overnight at 4°C. After incubation, the beads were washed five times with lysis buffer and eluted with SDS-PAGE loading buffer. Western blot analysis was performed using the anti-*Gl*Slt2 and anti-*Gl*Myb antibodies.

### Yeast two-hybrid assay

For Y2H screening, the full-length *GlSlt2* gene was inserted into the pGBKT7 bait vector. The WT mycelium used for generating the cDNA library was collected after being grown in a medium with glucose as the sole carbon source at 28°C in the dark for 7 days. The bait and the cDNA library were co-transformed into the Y2HGold yeast strain. After culturing on synthetic medium lacking Trp and Leu (SD/-Trp/-Leu) for 2 days at 30°C, transformants were plated on SD/-Ade-His-Leu-Trp medium containing X-α-Dgalactoside (X-α-gal) for 2 days.

For further validation, the full-length *GlMyb* gene and truncated versions (*GlMyb*^1–163^, *GlMyb*^164–629^) were individually cloned into the pGADT7 prey vector. These constructs were co-transformed into yeast cells and grown on SD/-Ade-His-Leu-Trp/X-α-Gal medium at 30°C for 2 days. The appearance of blue colonies on the plates signified positive protein-protein interactions.

### BiFC assay

For the BiFC assay, the recombinant constructs PVN-Slt2 were co-transformed with PVC-Myb, PVC-Myb^1–163^, and pVC-Myb^164–629^ into the yeast strain SFY2620, and cultured in liquid SD medium lacking Leu and Ura (SD/-Leu-Ura) at 30°C for 3 days. Subsequently, they were photomicrographed with a BX53 DIC/BF Olympus confocal microscopy with DP27 digital camera (Shinjuku, Tokyo, Japan). DIC indicates yeast cell morphology under the normal white field of view; Venus, a variant of GFP, yeast cell morphology under the green fluorescence.

### Western blotting

Proteins (25 µg) from different strains were resolved using 12% SDS-PAGE for immunoblotting. The primary antibodies used and their dilutions were as follows: rabbit anti-p44/42 MAPK (Erk1/2) antibody (anti-*Gl*Slt2, 1:1,000, Cell Signaling Technology, Cat# 9102), Phospho-p44/42 MAPK (Erk1/2) antibody (anti-P-*Gl*Slt2, 1:1,000, Cell Signaling Technology, Cat# 4376), rabbit anti-phospho Ser/Thr antibody (1:1,000, Abcam, Cat# ab117253), and mouse anti-actin (1:1,000, M20011; Abcam).

### *In vivo* phosphorylation assays

All strains were collected from the liquid culture medium with microcrystalline cellulose as the sole carbon source. The mycelia of all strains were snap-frozen in liquid N2, and protein extraction was performed as detailed by Zhang et al. (62). Subsequently, proteins from all different strains were immunoprecipitated using an anti-*Gl*Myb antibody. The precipitated proteins were then resolved on a 12% SDS-PAGE gel for Western blot analysis with either anti-phospho Ser/Thr or anti-*Gl*Myb antibodies.

### Purification of recombinant proteins and antibody generation

The vectors pColdI-Slt2, pColdI-Slt2^T191D/Y193D^, pColdI-Myb, pColdI-Myb^S245A^, and pColdI-Myb^S245D^ were introduced into *E. coli* strain BL21 (DE3). Induction of protein expression was achieved with 500 µM isopropyl-β-D-thiogalactopyranoside (IPTG), and cultures were incubated for 16 h at 16°C. Recombinant protein purification was carried out using nickel-nitrilotriacetate (Ni-NTA) agarose columns (Sangon, C600033). The purified His-Myb protein was dispatched to Chemgen Biotech for rabbit immunization and antibody generation.

### *In vitro* phosphorylation assay

*In vitro* phosphorylation assays were conducted following established protocols (63). Briefly, His-Slt2 or His-Slt2^T191D/Y193D^ protein (3 µg) was mixed with His-Myb or His-Myb^S245A^ protein (1 µg) in a kinase buffer containing 20 mM Tris-HCl buffer, 100 mM NaCl, 20 mM MgCl_2_, 2 mM DTT, and 10 mM ATP for 30 min at 30°C, in a total volume of 50 µL. For dephosphorylation with calf intestinal alkaline phosphatase (CIAP, Thermo Fisher), 1 µL of CIAP was added to 25 µL of the kinase assay mix and incubated at 37°C for an additional 30 min. The samples were then resolved by 12% SDS–PAGE and analyzed using an anti-phospho Ser/Thr antibody.

### Yeast one-hybrid assay

The constructed recombinant plasmids (pAbAi-*CBH1*, *CBH2*, *CBH3*, *EG1*, *EG2*, *EG3*, *EG4*, and *EG5*) were digested with *Bst*BI to linearize and then transformed into Y1HGold yeast strains. These strains were cultivated in synthetic medium lacking Ura (SD/-Ura) at 30°C for 3 days. Subsequently, pGADT7-Myb and the empty vector control (pGADT7) were introduced into the Y1HGold yeast strains and grown in SD medium lacking Leu (SD/-Leu) at 30°C for 3 days with different concentrations of aureobasidin A (AbA).

### EMSA assay

The EMSA was performed using the EMSA kit (Beyotime, Shanghai, China) according to the instructions. The His-Myb, His-Myb^S245A^, and His-Myb^S245D^ proteins with biotin-labeled probes, biotin-labeled mutant probes, or unlabeled competitive probes at 25°C for 20 min according to the manufacturer’s instructions. A control lacking protein was also prepared. These mixtures were then separated on a 6% nondenaturing polyacrylamide gel. After electrophoresis, the gels were transferred to membranes and imaged using a chemiluminescent nucleic acid detection kit from Beyotime, Shanghai, China, with the Bio-Rad ChemiDoc Touch system for color development.

The supershift assays were conducted based on previous research (64). The His-Myb, His-Myb^S245A^, and His-Myb^S245D^ proteins were incubated with His-Slt2^T191D/Y193D^ in kinase assay buffer at 30°C for 30 min. After reactions, the mixtures, as well as His-Myb, His-Myb^S245A^, His-Myb^S245D^, or His-Slt2^T191D/Y193D^ protein, were added with biotin-labeled probes for EMSA assays. The Image J software was used to quantify the gel band intensities.

### ChIP-quantitative PCR (ChIP-qPCR) assays

ChIP assays were performed as described previously (65). Briefly, 1-week-old mycelia (2 g) collected from the liquid culture medium with microcrystalline cellulose as the sole carbon source, with or without 20 µM PD98059 treatment, were cross-linked with 1% formaldehyde for protein-DNA fixation. The samples were ground, and the chromatin was extracted with anti-*Gl*Myb antibody. The protein A-agarose beads were used for purifying the DNA-histone-antibody complex. Finally, the enriched DNA fragments were analyzed by qPCR. All primers used are listed in Table S2.

### Statistical analysis

Data analysis was conducted using GraphPad Prism 8, based on a minimum of three independent samples. The error bars represent the standard deviation (SD) of the triplicate measurements. One-way or two-way analysis of variance (ANOVA) was utilized to assess mean differences between groups with GraphPad Prism. Different letters correspond to *P* < 0.05.

## Data Availability

The data that support the findings of this study are available from the corresponding author upon reasonable request.

## SUPPLEMENTAL MATERIAL

Supplemental Figures and Tables mbio.01812-25-s0001.docx Fig. S1 to S8; Tables S1 to S2.

Supplemental Data mbio.01812-25-s0002.xlsx All numerical data underlying the figures and supplemental figures.

ASM does not own the copyrights to Supplemental Material that may be linked to, or accessed through, an article. The authors have granted ASM a non-exclusive, world-wide license to publish the Supplemental Material files. Please contact the corresponding author directly for reuse.

## References

[1] Baldrian P, Valásková V. 2008. Degradation of cellulose by basidiomycetous fungi. FEMS Microbiol Rev 32:501–521. doi:10.1111/j.1574-6976.2008.00106.x 18371173

[2] Lynd LR, Weimer PJ, van Zyl WH, Pretorius IS. 2002. Microbial cellulose utilization: fundamentals and biotechnology. Microbiol Mol Biol Rev 66:506–577, doi:10.1128/MMBR.66.3.506-577.2002 12209002 PMC120791

[3] Lu Y, Mehling M, Huan SQ, Bai L, Rojas OJ. 2024. Biofabrication with microbial cellulose: from bioadaptive designs to living materials. Chem Soc Rev 53:7363–7391. doi:10.1039/d3cs00641g 38864385

[4] Yan S, Xu Y, Yu XW. 2023. Role of cellulose response transporter-like protein CRT2 in cellulase induction in Trichoderma reesei. Biotechnol Biofuels Bioprod 16:118. doi:10.1186/s13068-023-02371-7 37488642 PMC10364367

[5] Glass NL, Schmoll M, Cate JHD, Coradetti S. 2013. Plant cell wall deconstruction by ascomycete fungi. Annu Rev Microbiol 67:477–498. doi:10.1146/annurev-micro-092611-150044 23808333

[6] Liao HP, Li SX, Wei Z, Shen QR, Xu YC. 2014. Insights into high-efficiency lignocellulolytic enzyme production by Penicillium oxalicum GZ-2 induced by a complex substrate. Biotechnol Biofuels 7. doi:10.1186/s13068-014-0162-2 PMC4239378 25419234

[7] Sorour AA, Olama ZA, El-Naggar MY, Ali SM. 2023. Bioprocess development for extraction and purification of cellulases from Aspergillus niger 3ASZ using statistical experimental design techniques. Int J Biol Macromol 242:124759. doi:10.1016/j.ijbiomac.2023.124759 37150365

[8] Zhang J, Zhang G, Wang W, Wang W, Wei D. 2018. Enhanced cellulase production in Trichoderma reesei RUT C30 via constitution of minimal transcriptional activators. Microb Cell Fact 17:75. doi:10.1186/s12934-018-0926-7 29773074 PMC5956553

[9] Hu YR, Dong HZ, Chen HL, Shen XY, Li HH, Wen Q, Wang FQ, Qi YC, Shen JW. 2024. PoSnf1 affects cellulose utilization through interaction with cellobiose transporter in Pleurotus ostreatus. Int J Biol Macromol 275:133503. doi:10.1016/j.ijbiomac.2024.133503 38944091

[10] Wang Q, Xiao TT, Juan JX, Qian WB, Zhang JJ, Chen H, Shen XF, Huang JC. 2023. Lignocellulose degradation efficiency of Agaricus bisporus strains grown on wheat straw-based compost. J Agric Food Chem 71:10607–10615. doi:10.1021/acs.jafc.3c02595 37417743

[11] Zied DC, da Silva Freitas MA, de Almeida Moreira BR, da Silva Alves L, Pardo-Giménez A. 2023. A comparative analysis of biodegradation and bioconversion of Lentinula edodes and other exotic mushrooms. Microorganisms 11:897. doi:10.3390/microorganisms11040897 37110320 PMC10142386

[12] Amore A, Giacobbe S, Faraco V. 2013. Regulation of cellulase and hemicellulase gene expression in fungi. Curr Genomics 14:230–249. doi:10.2174/1389202911314040002 24294104 PMC3731814

[13] Ilmén M, Saloheimo A, Onnela ML, Penttilä ME. 1997. Regulation of cellulase gene expression in the filamentous fungus Trichoderma reesei. Appl Environ Microbiol 63:1298–1306. doi:10.1128/aem.63.4.1298-1306.1997 9097427 PMC168424

[14] Filho JAF, Rosolen RR, Almeida DA, de Azevedo PHC, Motta MLL, Aono AH, dos Santos CA, Horta MAC, de Souza AP. 2021. Trends in biological data integration for the selection of enzymes and transcription factors related to cellulose and hemicellulose degradation in fungi. 3 Biotech 11. doi:10.1007/s13205-021-03032-y PMC8548487 34777932

[15] Hasper AA, Trindade LM, van der Veen D, van Ooyen AJJ, de Graaff LH. 2004. Functional analysis of the transcriptional activator XlnR from Aspergillus niger. Microbiology (Reading, Engl) 150:1367–1375. doi:10.1099/mic.0.26557-0 15133098

[16] Raulo R, Kokolski M, Archer DB. 2016. The roles of the zinc finger transcription factors XlnR, ClrA and ClrB in the breakdown of lignocellulose by Aspergillus niger. AMB Express 6:5. doi:10.1186/s13568-016-0177-0 26780227 PMC4715039

[17] Havukainen S, Valkonen M, Koivuranta K, Landowski CP. 2020. Studies on sugar transporter CRT1 reveal new characteristics that are critical for cellulase induction in Trichoderma reesei. Biotechnol Biofuels 13:158. doi:10.1186/s13068-020-01797-7 32944074 PMC7491124

[18] Znameroski EA, Li X, Tsai JC, Galazka JM, Glass NL, Cate JHD. 2014. Evidence for transceptor function of cellodextrin transporters in Neurospora crassa. J Biol Chem 289:2610–2619. doi:10.1074/jbc.M113.533273 24344125 PMC3908395

[19] Gustin MC, Albertyn J, Alexander M, Davenport K. 1998. MAP kinase pathways in the yeast Saccharomyces cerevisiae. Microbiol Mol Biol Rev 62:1264–1300. doi:10.1128/MMBR.62.4.1264-1300.1998 9841672 PMC98946

[20] Hohmann S. 2002. Osmotic stress signaling and osmoadaptation in yeasts. Microbiol Mol Biol Rev 66:300–372. doi:10.1128/MMBR.66.2.300-372.2002 12040128 PMC120784

[21] Hamel LP, Nicole MC, Duplessis S, Ellis BE. 2012. Mitogen-activated protein kinase signaling in plant-interacting fungi: distinct messages from conserved messengers. Plant Cell 24:1327–1351. doi:10.1105/tpc.112.096156 22517321 PMC3398478

[22] Ahmadpour D, Maciaszczyk-Dziubinska E, Babazadeh R, Dahal S, Migocka M, Andersson M, Wysocki R, Tamás MJ, Hohmann S. 2016. The mitogen-activated protein kinase Slt2 modulates arsenite transport through the aquaglyceroporin Fps1. FEBS Lett 590:3649–3659. doi:10.1002/1873-3468.12390 27607883

[23] Ai WD, Bertram PG, Tsang CK, Chan TF, Zheng XFS. 2002. Regulation of subtelomeric silencing during stress response. Mol Cell 10:1295–1305. doi:10.1016/S1097-2765(02)00695-0 12504006

[24] Pujol-Carrion N, Pavón-Vergés M, Arroyo J, de la Torre-Ruiz MA. 2021. The MAPK Slt2/Mpk1 plays a role in iron homeostasis through direct regulation of the transcription factor Aft1. Biochim Biophys Acta Mol Cell Res 1868:118974. doi:10.1016/j.bbamcr.2021.118974 33549702

[25] Jung US, Sobering AK, Romeo MJ, Levin DE. 2002. Regulation of the yeast Rlm1 transcription factor by the Mpk1 cell wall integrity MAP kinase. Mol Microbiol 46:781–789. doi:10.1046/j.1365-2958.2002.03198.x 12410835

[26] Wang MY, Dong YM, Zhao QS, Wang FZ, Liu KM, Jiang BJ, Fang X. 2014. Identification of the role of a MAP kinase Tmk2 in Hypocrea jecorina (Trichoderma reesei). Sci Rep 4. doi:10.1038/srep06732 PMC4206845 25339247

[27] Feller A, Machemer K, Braun EL, Grotewold E. 2011. Evolutionary and comparative analysis of MYB and bHLH plant transcription factors. Plant J 66:94–116. doi:10.1111/j.1365-313X.2010.04459.x 21443626

[28] Stringlis IA, Yu K, Feussner K, de Jonge R, Van Bentum S, Van Verk MC, Berendsen RL, Bakker P, Feussner I, Pieterse CMJ. 2018. MYB72-dependent coumarin exudation shapes root microbiome assembly to promote plant health. Proc Natl Acad Sci USA 115:E5213–E5222. doi:10.1073/pnas.1722335115 29686086 PMC5984513

[29] Schmidt R, Schippers JHM, Mieulet D, Obata T, Fernie AR, Guiderdoni E, Mueller-Roeber B. 2013. MULTIPASS, a rice R2R3-type MYB transcription factor, regulates adaptive growth by integrating multiple hormonal pathways. Plant J 76:258–273. doi:10.1111/tpj.12286 23855375

[30] Liu ZQ, Deng B, Yuan H, Zhang BF, Liu JY, Meng JL, Chang MC. 2022. Transcription factor FfMYB15 regulates the expression of cellulase gene FfCEL6B during mycelial growth of Flammulina filiformis. Microb Cell Fact 21. doi:10.1186/s12934-022-01932-z PMC9578197 36253826

[31] Liu GZ, Li Q, Shang N, Huang JW, Ko TP, Liu WD, Zheng YY, Han X, Chen Y, Chen CC, Jin J, Guo RT. 2016. Functional and structural analyses of a 1,4-β-endoglucanase from Ganoderma lucidum. Enzyme Microb Technol 86:67–74. doi:10.1016/j.enzmictec.2016.01.013 26992795

[32] Sitarz AK, Mikkelsen JD, Højrup P, Meyer AS. 2013. Identification of a laccase from Ganoderma lucidum CBS 229.93 having potential for enhancing cellulase catalyzed lignocellulose degradation. Enzyme Microb Technol 53:378–385. doi:10.1016/j.enzmictec.2013.08.003 24315640

[33] Chen SL, Xu J, Liu C, Zhu YJ, Nelson DR, Zhou SG, Li CF, Wang LZ, Guo X, Sun YZ, et al.. 2012. Genome sequence of the model medicinal mushroom Ganoderma lucidum. Nat Commun 3. doi:10.1038/ncomms1923 PMC3621433 22735441

[34] Lian LD, Shi LY, Zhu J, Liu R, Shi L, Ren A, Yu HS, Zhao MW. 2022. GlSwi6 positively regulates cellulase and xylanase activities through intracellular Ca^2+^ signaling in Ganoderma lucidum. J Fungi (Basel) 8:187. doi:10.3390/jof8020187 35205940 PMC8877461

[35] Shangguan JL, Qiao JJ, Liu H, Zhu L, Han XF, Shi L, Zhu J, Liu R, Ren A, Zhao MW. 2024. The CBS/H2S signalling pathway regulated by the carbon repressor CreA promotes cellulose utilization in Ganoderma lucidum. Commun Biol 7. doi:10.1038/s42003-024-06180-y PMC11024145 38632386

[36] Hu Y, Xu W, Hu S, Lian L, Zhu J, Shi L, Ren A, Zhao M. 2020. In Ganoderma lucidum, Glsnf1 regulates cellulose degradation by inhibiting GlCreA during the utilization of cellulose. Environ Microbiol 22:107–121. doi:10.1111/1462-2920.14826 31608522

[37] Zhang G, Sun ZH, Ren A, Shi L, Shi DK, Li XB, Zhao MW. 2017. The mitogen-activated protein kinase GlSlt2 regulates fungal growth, fruiting body development, cell wall integrity, oxidative stress and ganoderic acid biosynthesis in Ganoderma lucidum. Fungal Genet Biol 104:6–15. doi:10.1016/j.fgb.2017.04.004 28435030

[38] Alessi DR, Cuenda A, Cohen P, Dudley DT, Saltiel AR. 1995. PD 098059 is a specific inhibitor of the activation of mitogen-activated protein kinase kinase in vitro and in vivo. J Biol Chem 270:27489–27494. doi:10.1074/jbc.270.46.27489 7499206

[39] Wang Z, Chen JH, Wang LS, Ding J, Zhao MW, Liu R. 2022. GlPP2C1 silencing increases the content of Ganodermalingzhi polysaccharide (GL-PS) and enhances Slt2 phosphorylation. J Fungi 8:949. doi:10.3390/jof8090949 PMC9506439 36135674

[40] Canagarajah BJ, Khokhlatchev A, Cobb MH, Goldsmith EJ. 1997. Activation mechanism of the MAP kinase ERK2 by dual phosphorylation. Cell 90:859–869. doi:10.1016/s0092-8674(00)80351-7 9298898

[41] González-Rubio G, Sellers-Moya Á, Martín H, Molina M. 2021. A walk-through MAPK structure and functionality with the 30-year-old yeast MAPK Slt2. Int Microbiol 24:531–543. doi:10.1007/s10123-021-00183-z 33993419

[42] Han X, Wang Z, Shi L, Zhu J, Shi L, Ren A, Zhao M. 2022. Phospholipase D and phosphatidic acid mediate regulation in the biosynthesis of spermidine and ganoderic acids by activating GlMyb in Ganoderma lucidum under heat stress. Environ Microbiol 24:5345–5361. doi:10.1111/1462-2920.16211 36111803

[43] Wang M, Zhang M, Li L, Dong Y, Jiang Y, Liu K, Zhang R, Jiang B, Niu K, Fang X. 2017. Role of Trichoderma reesei mitogen-activated protein kinases (MAPKs) in cellulase formation. Biotechnol Biofuels 10:99. doi:10.1186/s13068-017-0789-x 28435444 PMC5397809

[44] Wang M, Zhao Q, Yang J, Jiang B, Wang F, Liu K, Fang X. 2013. A mitogen-activated protein kinase Tmk3 participates in high osmolarity resistance, cell wall integrity maintenance and cellulase production regulation in Trichoderma reesei. PLoS One 8:e72189. doi:10.1371/journal.pone.0072189 23991059 PMC3753334

[45] Huberman LB, Coradetti ST, Glass NL. 2017. Network of nutrient-sensing pathways and a conserved kinase cascade integrate osmolarity and carbon sensing in Neurospora crassa. Proc Natl Acad Sci USA 114:E8665–E8674. doi:10.1073/pnas.1707713114 28973881 PMC5642704

[46] Chen DD, Shi L, Yue SN, Zhang TJ, Wang SL, Liu YN, Ren A, Zhu J, Yu HS, Zhao MW. 2019. The Slt2-MAPK pathway is involved in the mechanism by which target of rapamycin regulates cell wall components in Ganoderma lucidum. Fungal Genet Biol 123:70–77. doi:10.1016/j.fgb.2018.12.005 30557614

[47] Madden K, Sheu YJ, Baetz K, Andrews B, Snyder M. 1997. SBF cell cycle regulator as a target of the yeast PKC-MAP kinase pathway. Science 275:1781–1784. doi:10.1126/science.275.5307.1781 9065400

[48] Ray A, Hector RE, Roy N, Song JH, Berkner KL, Runge KW. 2003. Sir3p phosphorylation by the Slt2p pathway effects redistribution of silencing function and shortened lifespan. Nat Genet 33:522–526. doi:10.1038/ng1132 12640455

[49] Watanabe Y, Irie K, Matsumoto K. 1995. Yeast RLM1 encodes a serum response factor-like protein that may function downstream of the Mpk1 (Slt2) mitogen-activated protein kinase pathway. Mol Cell Biol 15:5740–5749. doi:10.1128/MCB.15.10.5740 7565726 PMC230825

[50] Kim WC, Kim JY, Ko JH, Kang H, Han KH. 2014. Identification of direct targets of transcription factor MYB46 provides insights into the transcriptional regulation of secondary wall biosynthesis. Plant Mol Biol 85:589–599. doi:10.1007/s11103-014-0205-x 24879533

[51] Ramalingam R, Ennis HL. 1997. Characterization of the Dictyostelium discoideum cellulose-binding protein CelB and regulation of gene expression. J Biol Chem 272:26166–26172. doi:10.1074/jbc.272.42.26166 9334183

[52] Roth SY, Denu JM, Allis CD. 2001. Histone acetyltransferases. Annu Rev Biochem 70:81–120. doi:10.1146/annurev.biochem.70.1.81 11395403

[53] Duch A, Canal B, Barroso SI, García-Rubio M, Seisenbacher G, Aguilera A, de Nadal E, Posas F. 2018. Multiple signaling kinases target Mrc1 to prevent genomic instability triggered by transcription-replication conflicts. Nat Commun 9:379. doi:10.1038/s41467-017-02756-x 29371596 PMC5785523

[54] Sanz AB, García R, Rodríguez-Peña JM, Nombela C, Arroyo J. 2018. Slt2 MAPK association with chromatin is required for transcriptional activation of Rlm1 dependent genes upon cell wall stress. Biochim Biophys Acta Gene Regul Mech 1861:1029–1039. doi:10.1016/j.bbagrm.2018.09.005 30343693

[55] Mu D, Shi L, Ren A, Li M, Wu F, Jiang A, Zhao M. 2012. The development and application of a multiple gene co-silencing system using endogenous URA3 as a reporter gene in Ganoderma lucidum. PLoS One 7:e43737. doi:10.1371/journal.pone.0043737 22937087 PMC3427163

[56] Gong T, Liao Y, He F, Yang Y, Yang DD, Chen XD, Gao XD. 2013. Control of polarized growth by the rho family GTPase RHO4 in budding yeast: requirement of the N-terminal extension of Rho4 and regulation by the Rho GTPase-activating protein Bem2. Eukaryot Cell 12:368–377. doi:10.1128/EC.00277-12 23264647 PMC3571307

[57] Sung MK, Huh WK. 2007. Bimolecular fluorescence complementation analysis system for in vivo detection of protein-protein interaction in Saccharomyces cerevisiae. Yeast 24:767–775. doi:10.1002/yea.1504 17534848

[58] Deplancke B, Dupuy D, Vidal M, Walhout AJM. 2004. A gateway-compatible yeast one-hybrid system. Genome Res 14:2093–2101. doi:10.1101/gr.2445504 15489331 PMC528925

[59] Wang Z, Qiu H, Li Y, Zhao M, Liu R. 2024. GlPRMT5 inhibits GlPP2C1 via symmetric dimethylation and regulates the biosynthesis of secondary metabolites in Ganoderma lucidum. Commun Biol 7:241. doi:10.1038/s42003-024-05942-y 38418849 PMC10902306

[60] Aqeel A, Ahmed Z, Akram F, Abbas Q. 2024. Cloning, expression and purification of cellobiohydrolase gene from Caldicellulosiruptor bescii for efficient saccharification of plant biomass. Int J Biol Macromol 271:132525. doi:10.1016/j.ijbiomac.2024.132525 38797293

[61] Randhawa A, Ogunyewo OA, Eqbal D, Gupta M, Yazdani SS. 2018. Disruption of zinc finger DNA binding domain in catabolite repressor Mig1 increases growth rate, hyphal branching, and cellulase expression in hypercellulolytic fungus Penicillium funiculosum NCIM1228. Biotechnol Biofuels 11:15. doi:10.1186/s13068-018-1011-5 29416560 PMC5784589

[62] Zhang ZY, Li JH, Li F, Liu HH, Yang WS, Chong K, Xu YY. 2017. OsMAPK3 phosphorylates OsbHLH002/OsICE1 and inhibits its ubiquitination to activate OsTPP1 and enhances rice chilling tolerance. Dev Cell 43:731–743. doi:10.1016/j.devcel.2017.11.016 29257952

[63] Shi L, Wang Z, Chen JH, Qiu H, Liu WD, Zhang XY, Martin FM, Zhao MW. 2024. LbSakA-mediated phosphorylation of the scaffolding protein LbNoxR in the ectomycorrhizal basidiomycete Laccaria bicolor regulates NADPH oxidase activity, ROS accumulation and symbiosis development. New Phytol 243:381–397. doi:10.1111/nph.19813 38741469

[64] Wu CJ, Shan W, Liu XC, Zhu LS, Wei W, Yang YY, Guo YF, Bouzayen M, Chen JY, Lu WJ, Kuang JF. 2022. Phosphorylation of transcription factor bZIP21 by MAP kinase MPK6-3 enhances banana fruit ripening. Plant Physiol 188:1665–1685. doi:10.1093/plphys/kiab539 34792564 PMC8896643

[65] Lian L, Shi L, Zhu J, Shi L, Ren A, You H, Liu R, Zhao M. 2022. GCN4 enhances the transcriptional regulation of AreA by interacting with SKO1 to mediate nitrogen utilization in Ganoderma lucidum. Appl Environ Microbiol 88:e0132222. doi:10.1128/aem.01322-22 36342130 PMC9680636

