# Supplementary material for: GlSlt2 positively regulates GlMyb-mediated cellulose utilization in Ganoderma lucidum
Source: mBio. 2025 Sep 8;16(10):e01812-25. doi: 10.1128/mbio.01812-25 (PMC12506057; doi:10.1128/mbio.01812-25)
Supplement: Supplemental Figures and Tables — Fig. S1 to S8; Tables S1 to S2. [file mbio.01812-25-s0001.docx]

Supplementary materials for

***Gl*Slt2 positively regulates *Gl*Myb-mediated cellulose utilization in *Ganoderma lucidum***

Zi Wang^1^, Yefan Li^1^, Hao Qiu^1^, Zhouyu Li^1^, Tianyu Ji^1^, Ang Ren^1^, Jing Zhu^1^, Liang Shi^1^, Mingwen Zhao*^1^, Rui Liu*^1^

^1^Key Laboratory of Agricultural Environmental Microbiology, Ministry of Agriculture and Rural Affairs; Microbiology Department, College of Life Sciences, Nanjing Agricultural University, Nanjing 210095, Jiangsu, P.R. China

*Corresponding author: Mingwen Zhao and Rui Liu. These authors contributed equally.

E-mail: ruiliu@njau.edu.cn

Tel: +0086-25-84395602, Fax: +0086-25-84395602


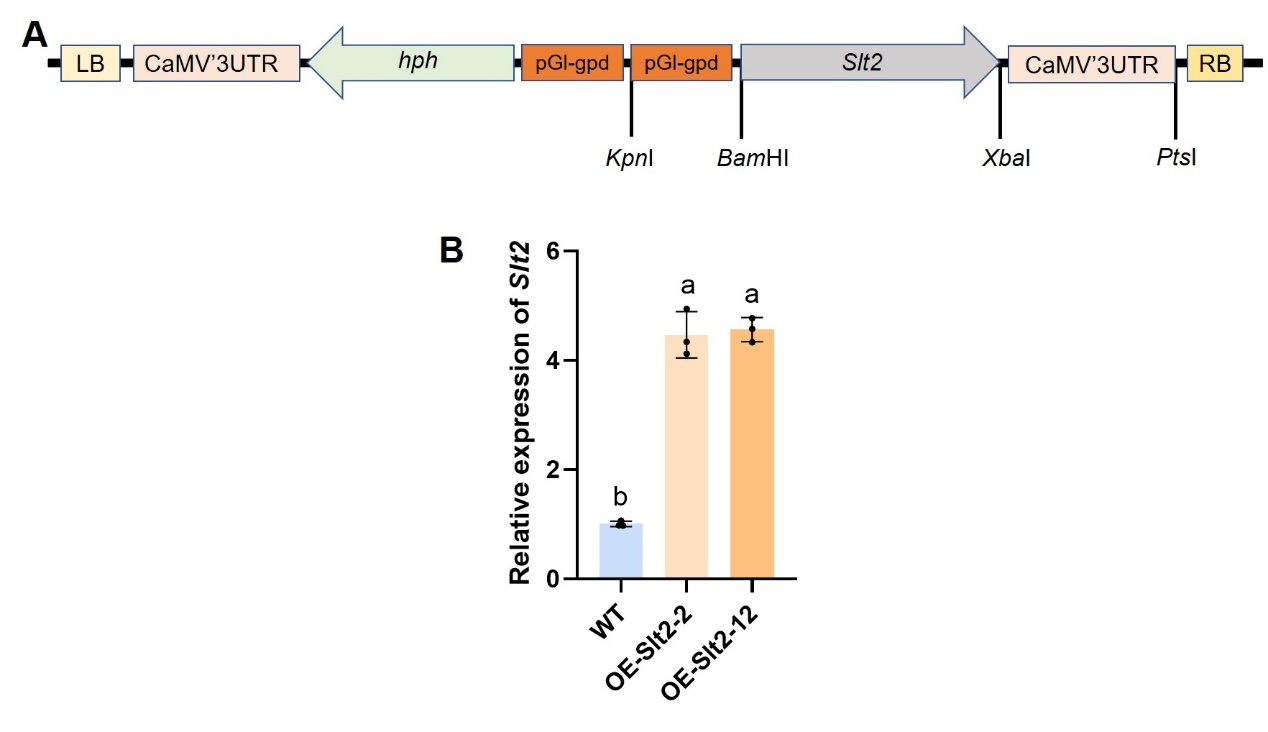


**Fig. S1** Construction of *GlSlt2*-overexpressing strains. (**A**) Construction of *GlSlt2*-overexpressing plasmids. In these plasmids, the transcription of *hph* and the target genes is driven by the *gpd* promoter. (**B**) Transcriptional levels of *GlSlt2* in different strains. The *GlSlt2* expression in the WT strain was deﬁned as 1.0. Data are presented as the mean ± SD (n = 3). Statistical significance is represented by different letters corresponding to *P* < 0.05 based on Tukey's multiple range test.


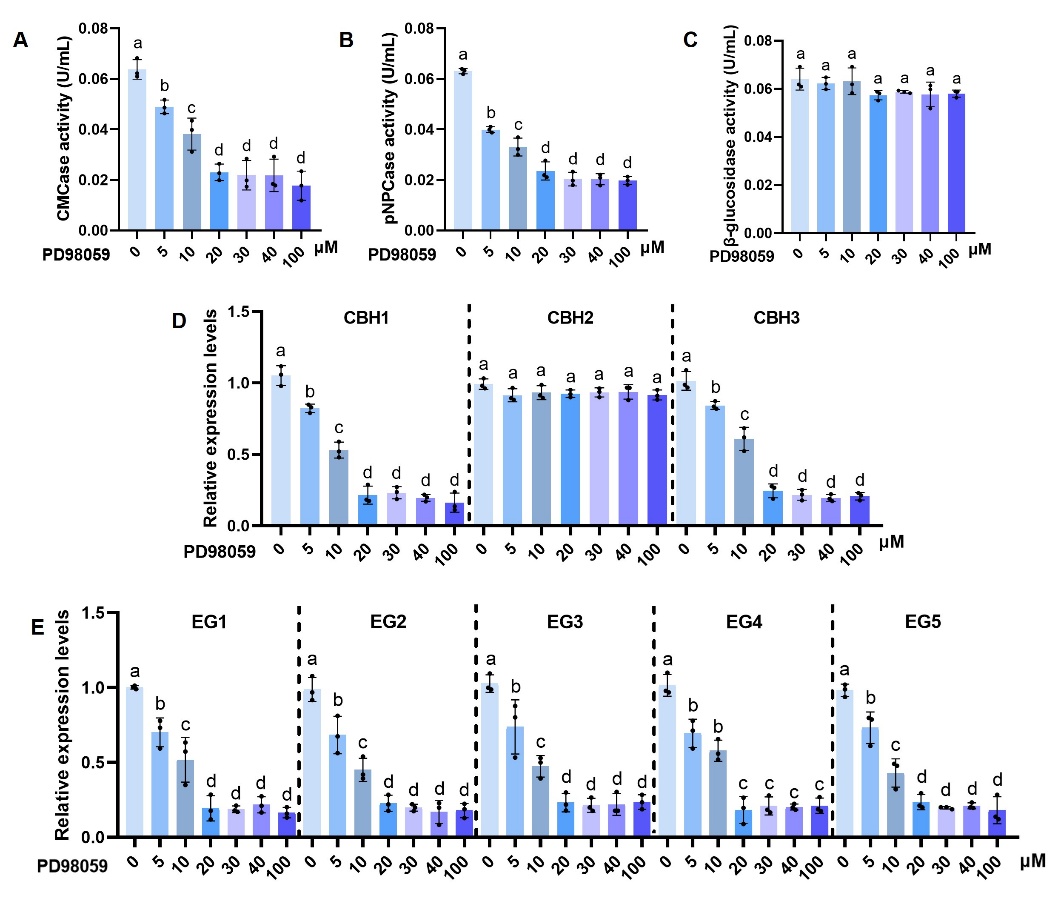


**Fig. S2** The impact of PD98059 on cellulose utilization. The endocellulase (**A**), exoglucanase (**B**) and β-glucosidase (**C**) activities were determined in the WT strains collected from the liquid culture medium with microcrystalline cellulose as the sole carbon source, containing different concentrations of PD98059. (**D-E**) The transcriptional levels of cellulase-related genes were measured in WT strains containing different concentrations of PD98059. Data are presented as the mean ± SD (n = 3). Statistical significance is represented by different letters corresponding to *P* < 0.05 based on Tukey's multiple range test.


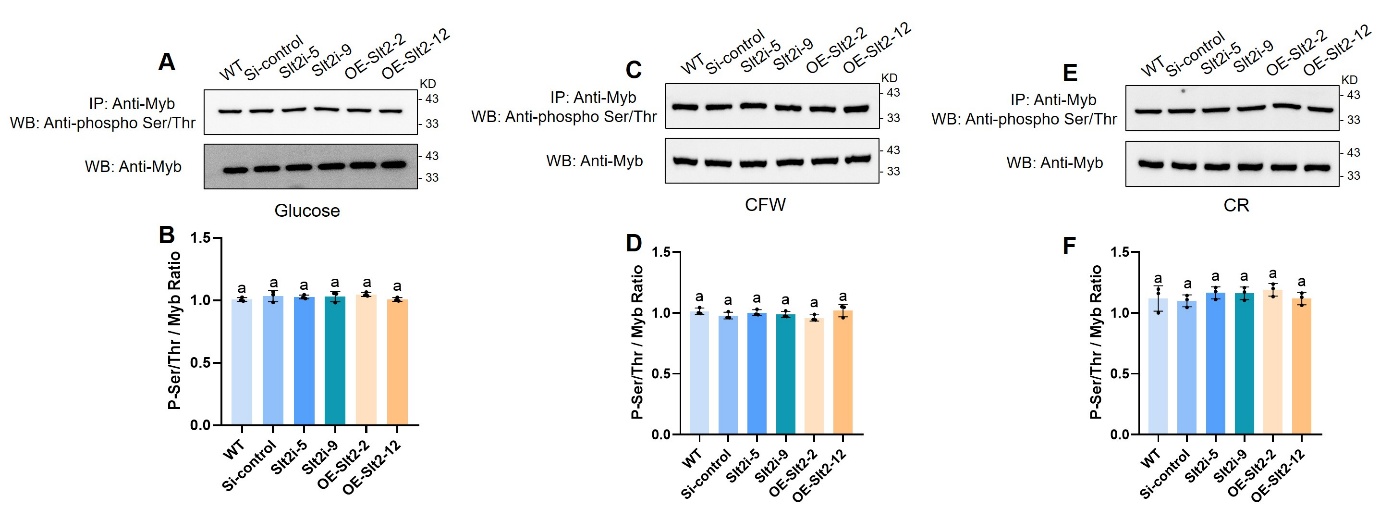


**Fig. S3** Detection of *Gl*Myb phosphorylation levels. (**A**) Detection of the phosphorylation level of *Gl*Myb in the WT, Si-control, *GlSlt2*-silenced and *GlSlt2*-overexpressing strains cultured on glucose as the sole carbon source medium by immunoblotting using an anti-phospho Ser/Thr antibody. (**B**) P-Ser/Thr / Myb ratio in panel (**A**). (**C**) Detection of the phosphorylation of *Gl*Myb in the WT, Si-control, *GlSlt2*-silenced and *GlSlt2*-overexpressing strains collected from the glucose-based medium with 1 mg/mL calcofluor white (CFW) by immunoblotting using an anti-phospho Ser/Thr antibody. (**D**) P-Ser/Thr / Myb ratio in panel (**C**). (**E**) Detection of the phosphorylation of *Gl*Myb in the WT, Si-control, *GlSlt2*-silenced and *GlSlt2*-overexpressing strains collected from the glucose-based medium with 4 mg/mL congo red (CR) by immunoblotting using an anti-phospho Ser/Thr antibody. (**F**) P-Ser/Thr / Myb ratio in panel (**E**). Data are presented as the mean ± SD (n = 3). Statistical significance is represented by different letters corresponding to *P* < 0.05 based on Tukey's multiple range test.


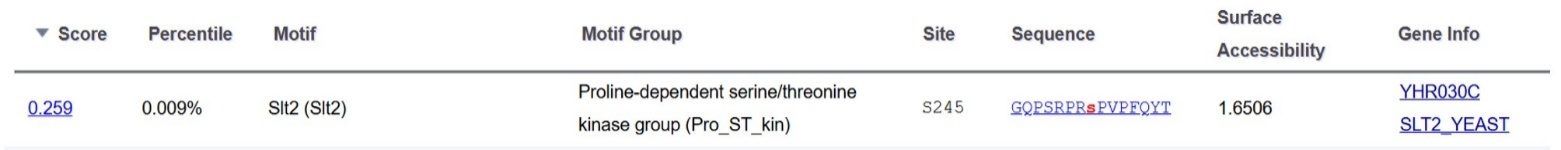


**Fig. S4** Prediction of phosphorylation sites in *Gl*Myb by Scansite software (<https://scansite4.mit.edu>).


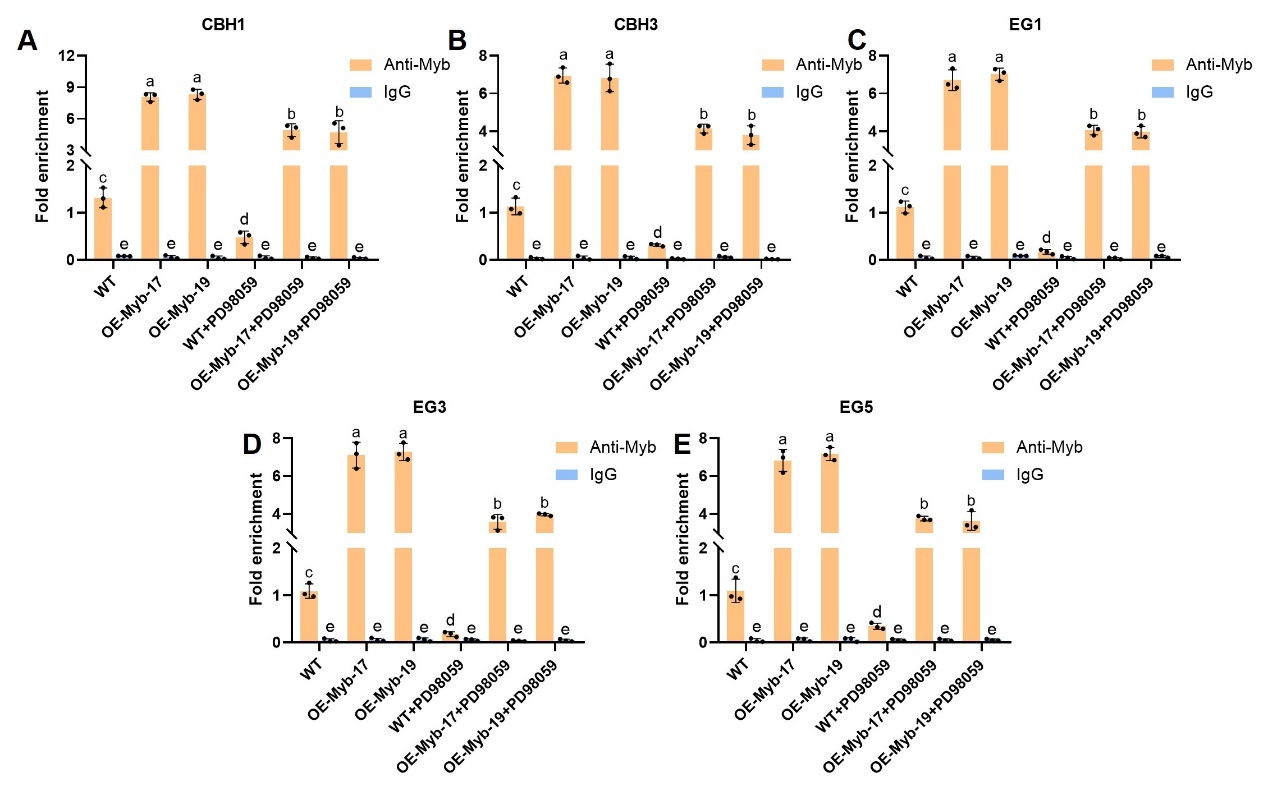


**Fig. S5** ChIP-qPCR analysis of the fold enrichment of cellulase-related genes. The WT and *GlMyb*-overexpressing strains were collected from the liquid culture medium with microcrystalline cellulose as the sole carbon source, with or without the addition of 20 μM PD98059. Subsequently, ChIP-qPCR was performed using the *Gl*Myb antibody. The fold enrichment of *CBH1* (**A**), *CBH3* (**B**), *EG1* (**C**), *EG3* (**D**) and *EG5* (**E**) in both WT and *GlMyb*-overexpressing strains, with or without the addition of 20 μM PD98059. The data in the WT strain was set as 1. Data are presented as the mean ± SD (n = 3). Statistical significance is represented by different letters corresponding to *P* < 0.05 based on Tukey's multiple range test.


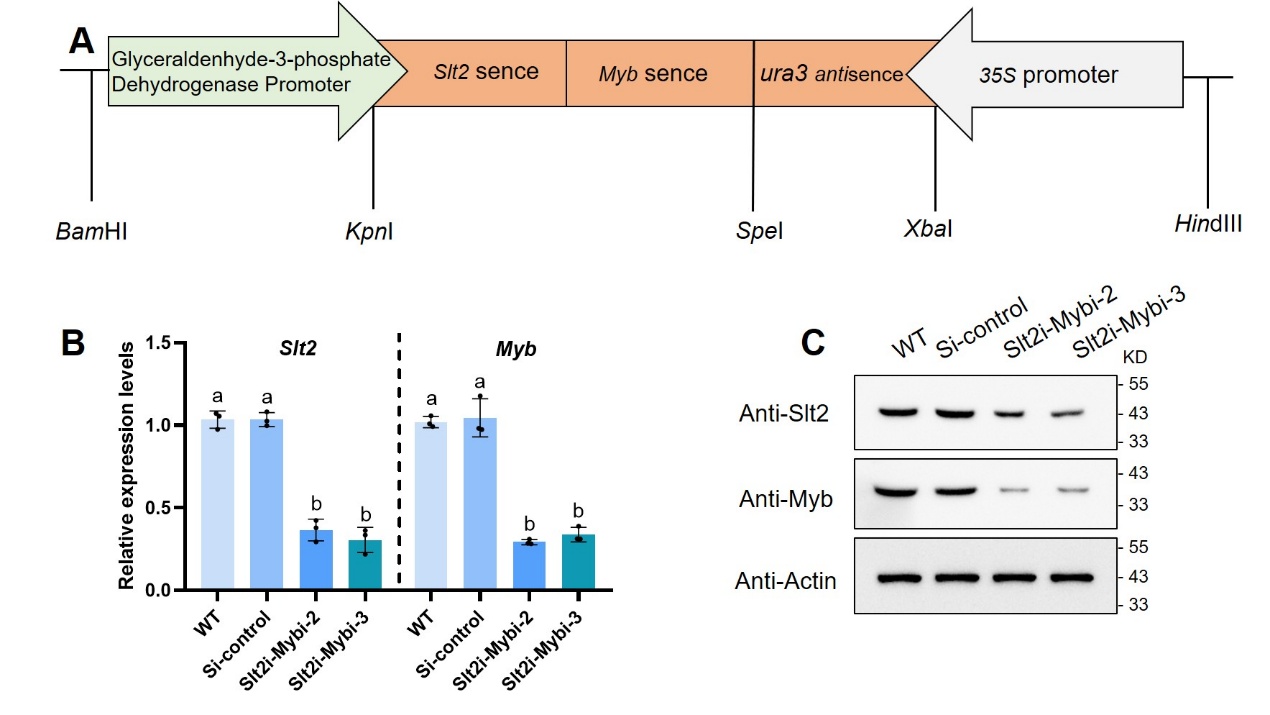


**Fig. S6** Construction of *GlSlt2*-*GlMyb* double-silenced strains. (**A**) Structure of the vector constructed for silencing the expression of *GlSlt2-GlMyb*. (**B**) qRT–PCR analysis of the expression of *GlSlt2* and *GlMyb* in the tested strains. (**C**) The *Gl*Slt2 and *Gl*Myb protein content in the WT, Si-control and *GlSlt2*-*GlMyb* double-silenced strains was detected by Western blot analysis. Data are presented as the mean ± SD (n = 3). Statistical significance is represented by different letters corresponding to *P* < 0.05 based on Tukey's multiple range test.


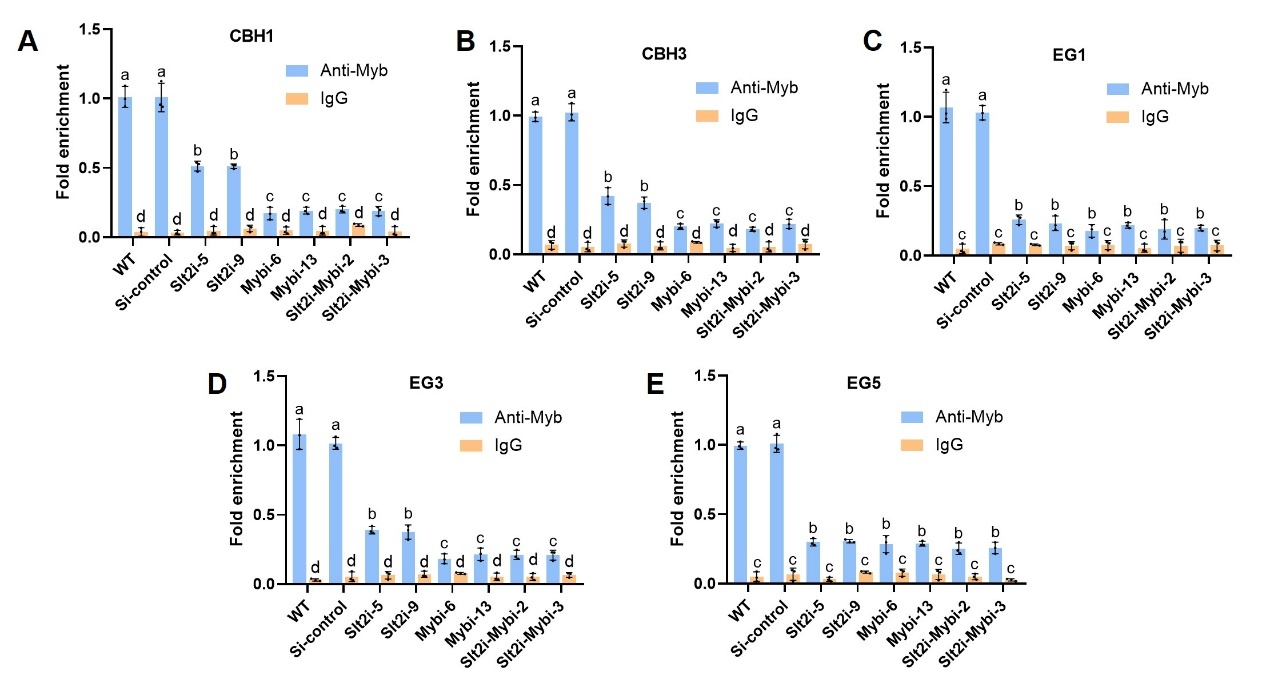


**Fig. S7** ChIP-qPCR analysis of the fold enrichment of cellulase-related genes. The WT, Si-control, *GlSlt2*-silenced, *GlMyb*-silenced, and *GlSlt2*-*GlMyb* double-silenced strains were collected from the liquid culture medium with microcrystalline cellulose as the sole carbon source. Subsequently, ChIP-qPCR was performed using the *Gl*Myb antibody. The fold enrichment of *CBH1* (**A**), *CBH3* (**B**), *EG1* (**C**), *EG3* (**D**) and *EG5* (**E**) among these strains. The data in the WT strain was set as 1. Data are presented as the mean ± SD (n = 3). Statistical significance is represented by different letters corresponding to *P* < 0.05 based on Tukey's multiple range test.


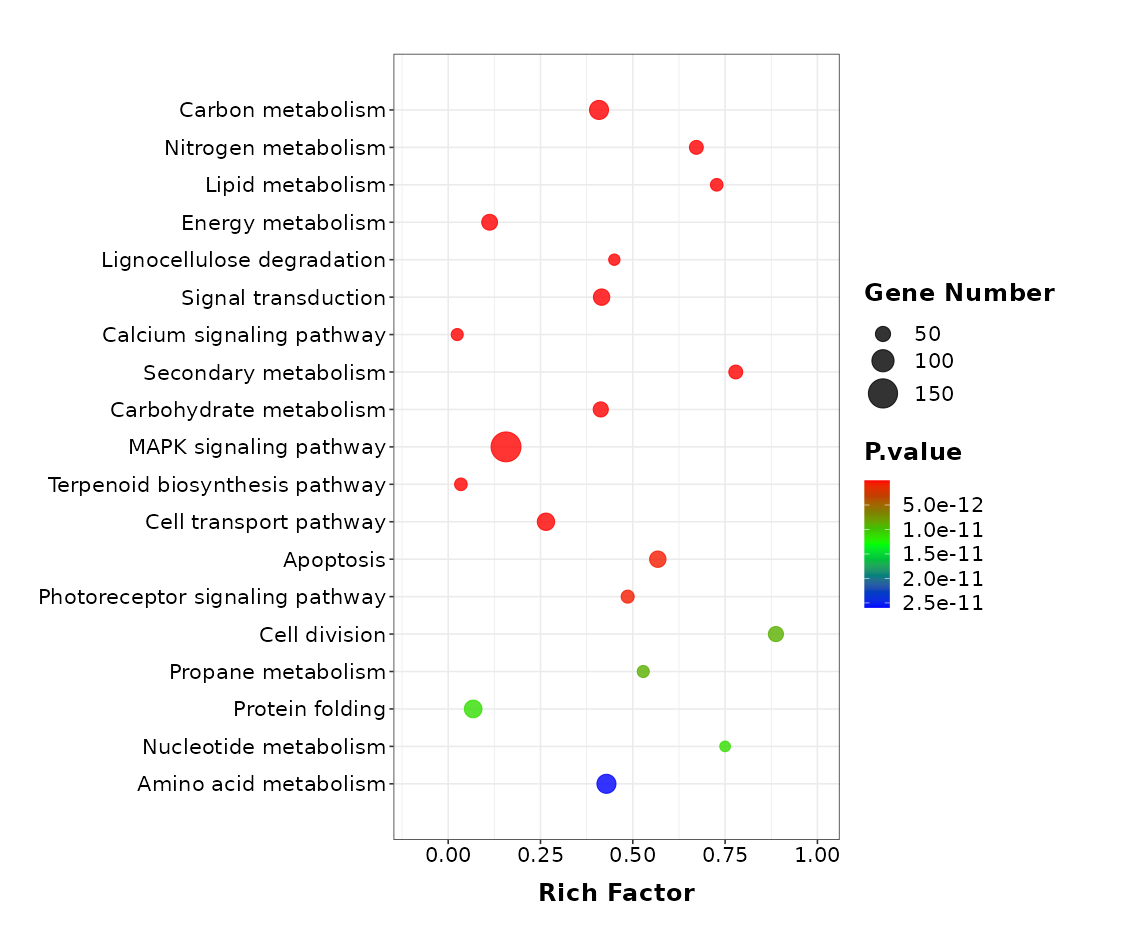


**Fig. S8** KEGG analysis of the distribution of *Gl*Myb binding motifs in the *G. lucidum* genome.

**Table S1.** Screening and identification of *Gl*Slt2-interacting proteins

| **Gene** | **Descriptions** |
| --- | --- |
| GL22843 | GTP-binding protein ypt1 |
| GL16044 | Iron reductase |
| GL22427 | PWI domain-containing protein |
| GL23262 | E2 ubiquitin-conjugating protein |
| GL22150 | Aldo/keto reductase |
| GL20421 | Cytochrome P450 |
| GL26736 | Small GTPase superfamily |
| GL24901 | Ary-alcohol dehydrogenase |
| GL24064 | Alpha/beta-hydrolase |
| GL30464 | Myb transcription factor |
| GL17949 | NAD(P)-binding protein |
| GL25243 | Arginine methyl transferase |
| GL24059 | 3-hydroxy-3-methylglutaryl coenzyme A reductase |

**Table S2.** Primers used in the experiment

| **Primers** | **Primer sequences (5′ to 3′)** | **Description** |
| --- | --- | --- |
| RT-18S-F | TATCGAGTTCTGACTGGGTTGT | Detects the *18S* expression |
| RT-18S-R | ATCCGTTGCTGAAAGTTGTAT |  |
| RT-GlSlt2-F | AAGAAGATCACGAACATCAACACCA | Detects the *GlSlt2* expression |
| RT-GlSlt2-R | AACCGCACAGGGTTTGGTAAATGAA |  |
| RT-GlCBH1-F | ACTGGACTGGCTCTGCTAACG | Detects the *GlCBH1* expression |
| RT-GlCBH1-R | ATCGCAGCCGTCCTTGTCG |  |
| RT-GlCBH2-F | ACGCTCACCACCGAGAC | Detects the *GlCBH2* expression |
| RT-GlCBH2-R | CGTAGTCAGCACCATCCAG |  |
| RT-GlCBH3-F | GCTTTGCCCTGACCCTGTC | Detects the *GlCBH3* expression |
| RT-GlCBH3-R | CGTAGGTGGTGTCATCCTGTGA |  |
| RT-GlEG1-F | CTCCGTCCAATTTGTCGT | Detects the *GlEG1* expression |
| RT-GlEG1-R | GTTCCACTGTTGTCGCTGT |  |
| RT-GlEG2-F | ACGGTTCAATGGCGAGATAA | Detects the *GlEG2* expression |
| RT-GlEG2-R | CGCACCTGCTTGACGAAT |  |
| RT-GlEG3-F | CACCGACTCACTCACCCTCA | Detects the *GlEG3* expression |
| RT-GlEG3-R | CGCGGACACCGTTCACA |  |
| RT-GlEG4-F | CGCGTCTGGCTCAGTTTG | Detects the *GlEG4* expression |
| RT-GlEG4-R | CAGCGTAGTATGGGCTCAAGT |  |
| RT-GlEG5-F | GTTCCTCGCTTTGTCAG | Detects the *GlEG5* expression |
| RT-GlEG5-R | CACGAATTCAGGGTCGC |  |
| OE-GlSlt2-F | GGATCCATGCAGCGACACTCCTTCA | For the construction of *GlSlt2* gene overexpression vector |
| OE-GlSlt2-R | TCTAGATCATCTCCGAATATGGG |  |
| GlMybi-F | GGTACCAGAATGCTGGAGCGAAGG | For the construction of *GlSlt2-GlMyb* double-silenced vector |
| GlMybi-R | ACTAGTCGTAGCCATGAGGATTAGGA |  |
| GlSlt2i-F | ACTGGTACCCGCTCTGGCGAAGGATGTG | For the construction of *GlSlt2-GlMyb* double-silenced vector |
| GlSlt2i-R | ACTAGTCAGGGTTTGGTAAATGAACGACT |  |
| RT-GlMyb-F | AGGCAGTAGCGTCTTTGGT | Detects the *GlMyb* expression |
| RT-GlMyb-R | GTTGCGGGCGATTATGGA |  |
| pAbAi-CBH1-F | CTATGTTACCCGTAGACTATGTTACCCGTAGACTATGTTACCCGTAGACC | For cloning the *CBH1* gene promoter into the pAbAi vector |
| ***Continued*** | | |
| **Primers** | **Primer sequences (5′ to 3′)** | **Description** |
| pAbAi-CBH1-R | TCGAGGTCTACGGGTAACATAGTCTACGGGTAACATAGTCTACGGGTAACATAGGTAC | For cloning the *CBH1* gene promoter into the pAbAi vector |
| pAbAi-CBH2-F | CGACTTTAGCCGGGAGCGACTTTAGCCGGGAGCGACTTTAGCCGGGAGCC | For cloning the *CBH2* gene promoter into the pAbAi vector |
| pAbAi-CBH2-R | TCGAGGCTCCCGGCTAAAGTCGCTCCCGGCTAAAGTCGCTCCCGGCTAAAGTCGGTAC |  |
| pAbAi-CBH3-F | CACGATTACCCGGGCGCACGATTACCCGGGCGCACGATTACCCGGGCGCC | For cloning the *CBH3* gene promoter into the pAbAi vector |
| pAbAi-CBH3-R | TCGAGGCGCCCGGGTAATCGTGCGCCCGGGTAATCGTGCGCCCGGGTAATCGTGGTAC |  |
| pAbAi-EG1-F | CCCCTGTTACCCGTGGTCCCTGTTACCCGTGGTCCCTGTTACCCGTGGTC | For cloning the *EG1* gene promoter into the pAbAi vector |
| pAbAi-EG1-R | TCGAGACCACGGGTAACAGGGACCACGGGTAACAGGGACCACGGGTAACAGGGGGTAC |  |
| pAbAi-EG2-F | CAACGTTCCCCTCTTTCAACGTTCCCCTCTTTCAACGTTCCCCTCTTTCC | For cloning the *EG2* gene promoter into the pAbAi vector |
| pAbAi-EG2-R | TCGAGGAAAGAGGGGAACGTTGAAAGAGGGGAACGTTGAAAGAGGGGAACGTTGGTAC |  |
| pAbAi-EG3-F | CTATAGTTACCCGTTCATATAGTTACCCGTTCATATAGTTACCCGTTCAC | For cloning the *EG3* gene promoter into the pAbAi vector |
| pAbAi-EG3-R | TCGAGTGAACGGGTAACTATATGAACGGGTAACTATATGAACGGGTAACTATAGGTAC |  |
| pAbAi-EG4-F | CAATATTCACCCGGCCAAATATTCACCCGGCCAAATATTCACCCGGCCAC | For cloning the *EG4* gene promoter into the pAbAi vector |
| pAbAi-EG4-R | TCGAGTGGCCGGGTGAATATTTGGCCGGGTGAATATTTGGCCGGGTGAATATTGGTAC |  |
| pAbAi-EG5-F | CCCAAGTTACGCGCCCACCAAGTTACGCGCCCACCAAGTTACGCGCCCAC | For cloning the *EG5* gene promoter into the pAbAi vector |
| pAbAi-EG5-R | TCGAGTGGGCGCGTAACTTGGTGGGCGCGTAACTTGGTGGGCGCGTAACTTGGGGTAC |  |
| ChIP-GlCBH1-F | CGGTTATTTCCCTACCATTGG | For ChIP-qPCR assays |
| ChIP-GlCBH1-R | CGTGAAGCGCTGCGCACATGA |  |
| ***Continued*** | | |
| **Primers** | **Primer sequences (5′ to 3′)** | **Description** |
| ChIP-GlCBH3-F | TCGCTCCGGGTCGCCGTATCC | For ChIP-qPCR assays |
| ChIP-GlCBH3-R | GCATTTGCGATATTTATAACC |  |
| ChIP-GlEG1-F | GTGTCCTGATGGCCCCGGTCA | For ChIP-qPCR assays |
| ChIP-GlEG1-R | GAAGGAGAGGTCGAGCGACGC |  |
| ChIP-GlEG3-F | TTCCAATGTTTTCGGCAGCCT | For ChIP-qPCR assays |
| ChIP-GlEG3-R | CTTGTCCTGATTACCTCGGAA |  |
| ChIP-GlEG5-F | GCCTGCTCCTCCGCGCTGCTG | For ChIP-qPCR assays |
| ChIP-GlEG5-R | GAGAGGGGGGATGAGAGGGAG |  |
